# Exploiting breakdown in nonhost effector–target interactions to boost host disease resistance

**DOI:** 10.1073/pnas.2114064119

**Published:** 2022-08-22

**Authors:** Hazel McLellan, Sarah E. Harvey, Jens Steinbrenner, Miles R. Armstrong, Qin He, Rachel Clewes, Leighton Pritchard, Wei Wang, Shumei Wang, Thomas Nussbaumer, Bushra Dohai, Qingquan Luo, Priyanka Kumari, Hui Duan, Ana Roberts, Petra C. Boevink, Christina Neumann, Nicolas Champouret, Ingo Hein, Pascal Falter-Braun, Jim Beynon, Katherine Denby, Paul R. J. Birch

**Affiliations:** ^a^Division of Plant Sciences, School of Life Science, University of Dundee (at James Hutton Institute), Invergowrie, Dundee DD2 5DA, United Kingdom;; ^b^Centre for Novel Agricultural Products, Department of Biology, University of York, York YO10 5DD, United Kingdom;; ^c^Life Sciences, University of Warwick, Coventry CV4 7AL, United Kingdom;; ^d^Justus Liebig Universität Giessen, JLU Institute of Phytopathology, Giessen, Hesse, Germany;; ^e^Department of Cell and Molecular Sciences, James Hutton Institute, Invergowrie, Dundee DD2 5DA, United Kingdom;; ^f^Information and Computational Sciences, James Hutton Institute, Invergowrie, Dundee DD2 5DA, United Kingdom;; ^g^Department of Microbiology & Plant Pathology, University of California, Riverside, CA 92521;; ^h^Institute of Network Biology, Helmholtz Zentrum Munchen, German Research Centre for Environmental Health, Munich, Germany;; ^i^Simplot Plant Sciences, J. R. Simplot Company, Boise, ID 83707

**Keywords:** host range, plant immunity, effector-triggered susceptibility, oomycete, plant–microbe interactions

## Abstract

Plants are resistant to most microbial species due to nonhost resistance (NHR), providing broad-spectrum and durable immunity. However, the molecular components contributing to NHR are poorly characterised. We address the question of whether failure of pathogen effectors to manipulate nonhost plants plays a critical role in NHR. RxLR (Arg-any amino acid-Leu-Arg) effectors from two oomycete pathogens, *Phytophthora infestans and Hyaloperonospora arabidopsidis*, enhanced pathogen infection when expressed in host plants (*Nicotiana benthamiana* and Arabidopsis, respectively) but the same effectors performed poorly in distantly related nonhost pathosystems. Putative target proteins in the host plant potato were identified for 64 *P*. infestans RxLR effectors using yeast 2-hybrid (Y2H) screens. Candidate orthologues of these target proteins in the distantly related non-host plant Arabidopsis were identified and screened using matrix Y2H for interaction with RxLR effectors from both *P*. infestans and *H*. *arabidopsidis*. Few *P*. infestans effector-target protein interactions were conserved from potato to candidate Arabidopsis target orthologues (cAtOrths). However, there was an enrichment of *H*. *arabidopsidis* RxLR effectors interacting with cAtOrths. We expressed the cAtOrth AtPUB33, which unlike its potato orthologue did not interact with *P*. *infestans* effector PiSFI3, in potato and Nicotiana benthamiana. Expression of AtPUB33 significantly reduced *P*. *infestans* colonization in both host plants. Our results provide evidence that failure of pathogen effectors to interact with and/or correctly manipulate target proteins in distantly related non-host plants contributes to NHR. Moreover, exploiting this breakdown in effector-nonhost target interaction, transferring effector target orthologues from non-host to host plants is a strategy to reduce disease.

## Significance

Plant nonhost resistance (NHR) prevents infection by all members of most microbial species, but its molecular mechanisms are not well understood. We found that effector proteins from the potato blight pathogen *Phytophthora infestans*, which enhance infection in host plants, fail to enhance susceptibility in nonhost *Arabidopsis*. These *P. infestans* effectors often failed to interact with *Arabidopsis* orthologs of their potato target proteins, whereas many interactions were detected between these *Arabidopsis* orthologs and effectors from its adapted pathogen *Hyaloperonospora arabidopsidis*. Thus, breakdown in effector–target interactions in distantly related nonhost plants is likely a key component of NHR. Importantly, we demonstrate that exploiting this breakdown and expressing nonhost target orthologs in host plants provide a strategy to prevent crop disease.

Plant pathogenic microorganisms infect their hosts by secretion of effector proteins that can act outside (apoplastic effectors) or within (cytoplasmic effectors) plant cells to suppress immunity (1, 2). Yet, most pathogenic microbes equipped with effectors are unable to infect the majority of plant species. Nonhost resistance (NHR) is the phenomenon whereby all genotypes of a particular plant species are resistant to all genotypes of a given pathogen species, and as such, it is expected to be both broad spectrum and durable (3). In general, NHR is thought to result from one or more of the following factors: 1) the existence of preformed physical or chemical barriers preventing the pathogen getting a foothold in the plant; 2) recognition of pathogen effector proteins by plant resistance (R) proteins, leading to strong effector-triggered immunity (ETI); or 3) the failure of pathogen effectors to suppress pathogen-associated molecular pattern–triggered immunity (PTI) (4). If one solely takes into account inducible plant immunity, a model proposed by Schulze-Lefert and Panstruga (5) suggests that evolutionary distance may influence the relative contributions of PTI and ETI to NHR. Their model proposes that in nonhost plants that are closely related to the host, pathogen effectors are predicted to retain their interactions and activities upon target proteins that are likely to be closely related at the sequence level to those in the host plant. Under such circumstances, failure of the pathogen to infect is more likely to be due to ETI (i.e., the recognition of effectors by conserved R proteins). Conversely, in plants more distantly related to the normal host of a pathogen, failure to colonize is more likely to be due to lack of PTI suppression. This could be due to an inability of pathogen effectors to interact with and/or correctly manipulate their evolutionarily less conserved targets, rendering them unable to suppress PTI, which restricts pathogen colonization(5, 6).

In the last decade, strides have been made in both the identification of pathogen effector repertoires through genome/transcriptome sequencing and searches for conserved motifs (7–10) and their cognate plant target proteins using various protein–protein interaction techniques (11). Indeed, it is possible to carry out high-throughput yeast two-hybrid (Y2H) screens, whereby a large complement of proteins of interest can be screened simultaneously against an array of potential targets (12). In this way, candidate cytoplasmic effector sets from adapted *Arabidopsis* pathogens (the oomycete *Hyaloperonospora arabidopsidis*, the bacterium *Pseudomonas syringae* pathovar *tomato*, and the fungus *Golovinomyces orontii* [*Go*]) were screened against >8,000 *Arabidopsis* proteins, identifying candidate host protein targets potentially involved in the defense response to these pathogens (13, 14). Interestingly, these screens revealed that some effectors from these three pathogens, representing different evolutionary kingdoms of life, apparently converged on common host protein targets, suggesting that there may be shared strategies to suppress or modify host processes (13, 14). In contrast to such large-scale screens, there is also a body of work with more in-depth analysis of how effectors from different pathogens suppress and/or manipulate host defenses (11, 15). Bacterial type III effectors often possess enzyme activity and target components of plant immunity, such as pattern recognition receptors (PRRs); coreceptors, like BAK1; and signaling components, such as BIK1 and mitogen-activated protein kinases (MAPKs) (16). Many of the fungal effectors whose mode of action is known are apoplastic proteins that inhibit plant proteases or have chitin binding activity (17). However, many fungal and oomycete cytoplasmic effectors target diverse pathways and processes from signal transduction to RNA processing and silencing, hormone signaling, and secretion, often altering posttranslational modifications, such as ubiquitination and phosphorylation (11). Some oomycete effectors have evolved to use the activity of their host targets for infection; such targets can be regarded as susceptibility (S) factors (11, 18).

In light of these developments, candidate cytoplasmic effectors from the potato late blight pathogen *Phytophthora infestans* and *Arabidopsis* pathogen *H. arabidopsidis* were studied to determine whether they displayed activity as virulence factors in both host and distantly related nonhost pathosystems. *P. infestans* and *H. arabidopsidis* are both oomycetes that express the cytoplasmic RxLR (Arg–any amino acid–Leu–Arg) motif containing effectors during host colonization, several of which have been functionally characterized (19–22). *P. infestans* infects potato, tomato, eggplant, and the model solanaceous plant *Nicotiana benthamiana* (23) but not the model plant *Arabidopsis*, a member of the Brassicaceae (24). *Arabidopsis* can be artificially made to allow *P. infestans* to complete its infection cycle in laboratory conditions but only where plant defenses have been suppressed by preinfection with *Albugo laibachii* (25, 26). Attempts to render *Arabidopsis* susceptible to *P. infestans* by genetic means have not been successful. In contrast, *H. arabidopsidis* infects *Arabidopsis* but not members of the Solanaceae (27). Understanding the mechanisms by which *Arabidopsis* and solanaceous plants are resistant to *P. infestans* and *H. arabidopsidis,* respectively, will unlock potential to exploit NHR for crop protection.

In this study, we expressed RxLR effectors from *H. arabidopsidis* and *P. infestans* in their nonhost plants *N. benthamiana* and *Arabidopsis*, respectively, to determine whether these effectors contribute to pathogen virulence in these plants. Y2H assays were employed to identify proteins (potential targets) in potato that interacted with a selection of 64 *P. infestans* (Pi) RxLR effectors. We then tested whether the effectors from *P. infestans* were able to retain interaction with candidate *Arabidopsis thaliana* orthologs (cAtOrths) of their potato host targets as well as identifying *H. arabidopsidis* RxLR effectors (HaRxLs) that were able to interact with these *Arabidopsis* proteins. Finally, we tested whether expression in wild-type *Solanaceae* plants of *P. infestans* “target orthologs” from the nonhost *Arabidopsis*, which evade interaction with PiRxLR effectors, reduced the capacity of *P. infestans* to colonize its hosts.

## Results

### RxLR Effectors Are Unable to Enhance Pathogen Colonization in Distantly Related Nonhost Plants.

**Fig. 1.**
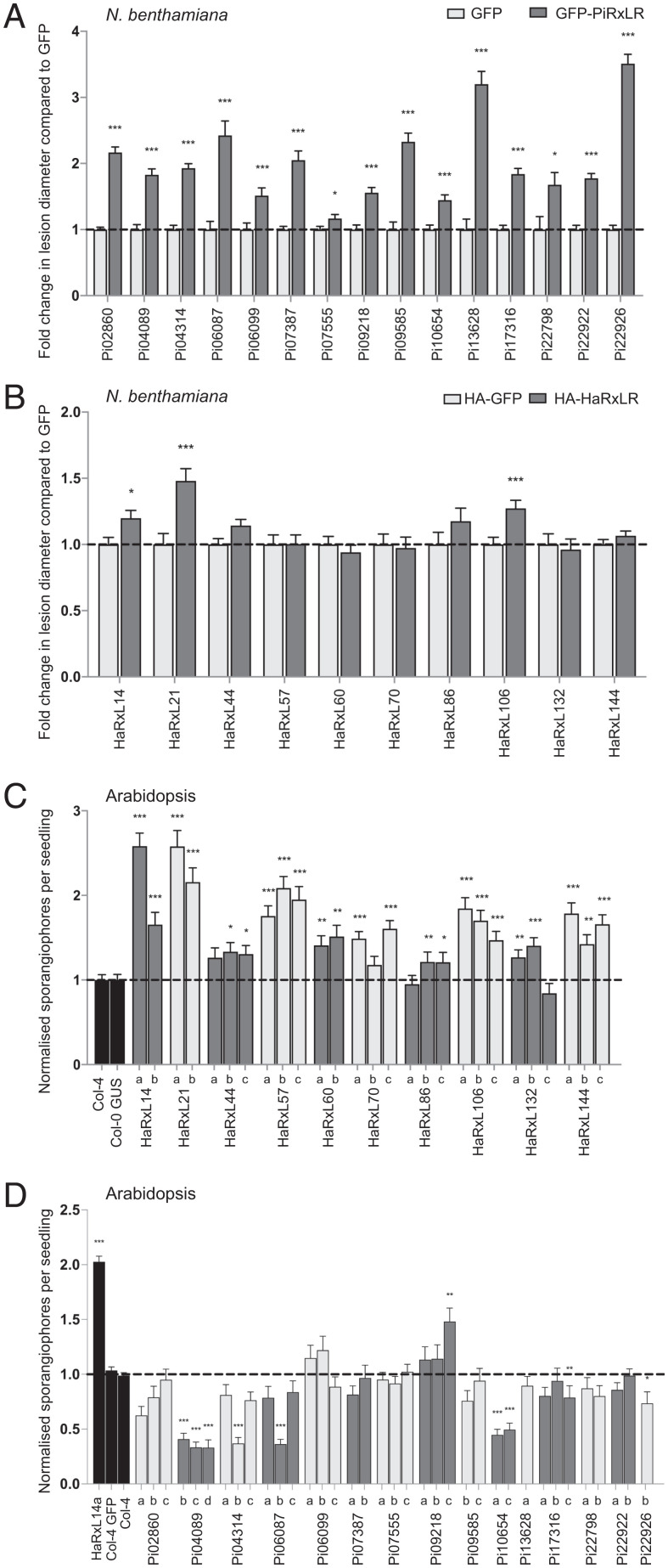
Effectors function poorly as virulence factors in a nonhost pathosystem. *Agrobacterium*-mediated transient expression of (*A*) *P. infestans* and (*B*) *H. arabidopsidis* (*Ha)* RxLR effectors in *N. benthamiana* followed by challenge with *P. infestans* and lesion diameter measurement at 7 days post-infection (dpi). Data for each effector are expressed as a fold change to the internal GFP or HA-GFP constructs, which were set to one. Sporangiophore counts per seedling at 4 dpi with *Hpa* of transgenic *Arabidopsis* expressing (*C*) HaRxL and (*D*) PiRxLR effectors under control of the Cauliflower mosaic virus (CaMV 35S) promoter. Data for two or three independent transgenic lines (indicated as a, b, and c) are shown for each effector. Sporangiophore counts are normalized to the counts of Columbia-4 (Col-4) wild type (WT) plants conducted at the same time with Col-4 set to one. Col-4 lines expressing GFP and Columbia-0 (Col-0) expressing ß-glucuronidase (GUS) were used as additional negative controls. A line expressing HaRxL14a was used in *D* as a susceptible control. Graphs show combined data from at least three independent replications of each experiment. Error bars are SE. Asterisks indicate significant differences as tested pairwise by the Student’s *t* test or the Mann–Whitney rank sum test. *P ≤ 0.05; **P ≤ 0.01; ***P ≤ 0.001.

To investigate whether effectors contribute to pathogenicity in both host and nonhost pathosystems, we selected RxLR effectors from two oomycete pathogens, *P. infestans* and *H. arabidopsidis*; expressed them individually in their host pathosystems (PiRxLRs in *N. benthamiana*; HaRxLs in *Arabidopsis*) and in their respective nonhost pathosystem (PiRxLRs in *Arabidopsis*; HaRxLs in *N. benthamiana*); and assessed whether expression enhanced colonization by host-adapted pathogens (*P. infestans* on *N. benthamiana* and *H. arabidopsidis* on *Arabidopsis*). Fifteen PiRxLR effectors were selected on the basis that they had been shown previously to enhance *P. infestans* colonization (28). They were expressed transiently (with green fluorescent protein (GFP) tags) in *N. benthamiana*, and plants were challenged 1 d later with *P. infestans* zoospores. Fig. 1*A* shows that as shown previously (28), when each of the PiRxLR effectors is expressed lacking their signal peptide, with N-terminal GFP tag, there is a significant increase in *P. infestans* lesion sizes, between 1.2- to >3.5-fold higher than controls expressing GFP alone as measured by lesion diameter. Each GFP–effector fusion protein was stable in planta (*SI Appendix*, Fig. S1*A*). We then expressed 10 HaRxLs in *N. benthamiana*. In contrast to the PiRxLRs, only three HaRxLs significantly enhanced *P. infestans* colonization of *N. benthamiana* compared with the GFP control, and those that reproducibly enhanced colonization were only modestly able to do so (between 1.2- and 1.5-fold) when compared with the majority *of P. infestans* effectors (Fig. 1*B*). All of those that failed to enhance *P. infestans* lesion sizes were nevertheless shown to be expressed as intact haemagglutinin (HA)-fusion proteins (*SI Appendix*, Fig. S1*B*). We did not observe any hypersensitive response (HR) in *N. benthamiana* in response to the HaRxL effector expression, so they are unlikely to be recognized by corresponding R proteins. The HaRxL effectors were selected on the basis of their ability to enhance *Arabidopsis* susceptibility to *H. arabidopsidis* when expressed in planta (refs. 19 and 29 and this study) rather than on similarity to other oomycete effectors at the sequence level. The few RxLR effectors that are conserved between *H. arabidopsidis* and *Phytophthora* species have been shown to suppress immunity in a range of host and nonhost plants (30, 31).

To perform reciprocal experiments, transgenic *Arabidopsis* plants individually expressing the 10 HaRxLs (*SI Appendix*, Fig. S2*A*) (lines are described in ref. 19) were challenged by *H. arabidopsidis*. At least two transgenic lines expressing each *H. arabidopsidis* effector were selected and shown to be significantly more susceptible to *H. arabidopsidis* infection as measured by sporangiophore counts, compared with control plants (Fig. 1*C*). In contrast, when transgenic *Arabidopsis* were generated expressing the 15 RxLR effectors from the nonhost pathogen *P. infestans* (*SI Appendix*, Fig. S2*B*), only one line expressing effector Pi09218 showed a significant enhancement in colonization by *H. arabidopsidis* compared with control plants, but this was not shared by the other two lines expressing this effector (Fig. 1*D*). By contrast, lines expressing two of the effectors (Pi04089 and Pi10654) showed significantly reduced *H. arabidopsidis* colonization, suggesting that activity of these effectors was detrimental to *H. arabidopsidis* infection. In order to understand this, we investigated whether these lines had an altered developmental phenotype. Lines expressing Pi04089 were found to display an early flowering phenotype (*SI Appendix*, Fig. S3), suggesting that overexpression of the *P. infestans* effector modifies this developmental process. Taken together, these data suggest that effectors perform better to enhance pathogen virulence in host plants rather than in a distantly related nonhost plant. This could be due to differences between each pathogen in their requirements for host manipulation to create a susceptible environment. However, it could also be because the effector has made an untargeted (or off-target) change to a host protein. Moreover, it could also be explained by failure of the effectors to either interact with or to appropriately manipulate the activities of target proteins in the nonhost plants. To explore the latter, we sought to identify interacting host potato proteins of *P. infestans* RxLR effectors.

### PiRxLR Effectors Interact with a Range of Host Proteins.

**Fig. 2.**
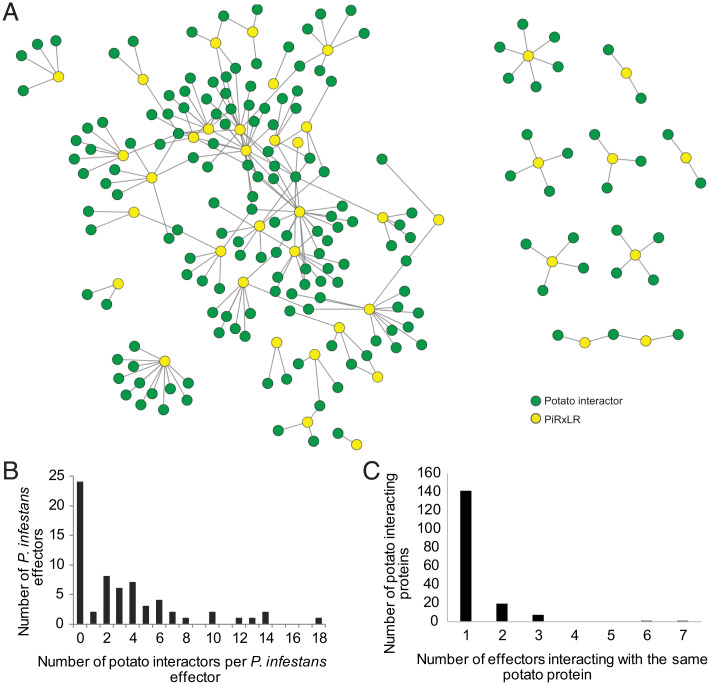
PiRXLR cY2H interaction network in potato. (*A*) Network diagram representation of the cY2H interaction network of 64 PiRxLR effectors (yellow circles) with 169 potato candidate target proteins (green circles). Straight edges indicate 215 protein–protein interactions. (*B*) Histogram showing the distribution of counts of PiRxLR effectors interacting with a given number of candidate target proteins (equivalent to the degree for each PiRxLR node in *A*). (*C*) Histogram showing the distribution of counts of candidate potato target proteins interacting with a given number of PiRXLR effectors (equivalent to the degree for each candidate target protein in *A*).

To identify candidate host targets of *P. infestans* effectors in potato, 64 PiRxLR effectors (Dataset S1), including the 15 in Fig. 1, were screened individually against a potato complementary DNA (cDNA) Y2H library (Dataset S2), here referred to as cY2H. This cY2H library was made from cDNA prepared from both compatible and incompatible potato–*P. infestans* interactions (32), and it has been extensively used to identify targets of PiRxLR effectors that have been verified in planta (32–44). The 64 PiRxLR effectors were prioritized based on being up-regulated during infection in a range of *P. infestans* genotypes, having diverse subcellular localizations, and possessing the capacity to enhance *P. infestans* colonization of *N. benthamiana* (28). Of the effectors screened, 24 (38%) did not reveal any interacting potato protein following the cY2H screens, despite a high number of yeast transformants (>1 × 10^6^) being obtained in each case. The remaining 40 effectors revealed a total of 169 interacting potato proteins (representing 215 interactions) (Fig. 2*A*, Dataset S2*A*, and *SI Appendix*, Fig. S4). Many effectors interacted with more than one potato protein (Fig. 2*B*), while a subset of potato proteins interacted with multiple effectors (Fig. 2*C* and Dataset S2*B*).

A full list of the RxLR effectors screened and the targets identified is shown in Dataset S2. Twenty-seven effectors (42%) shared a subset of their interacting host protein candidate targets with other effectors (Fig. 2, Dataset S2*B*, and *SI Appendix*, Fig. S4). The sequence similarity between PiRxLR effectors has been investigated previously using Markov clustering (MCL) (8), prompting us to see whether PiRxLRs with common host interactors were related at the primary sequence level. Little evidence was found for this; only 6 of the 27 effectors that shared host protein targets were from the same PiRxLR families (Pi17309 and Pi17316 in RxLRfam1, Pi16663 and Pi22922 in RxLRfam2, Pi21388 and PiIPIO4 in RxLRfam54) (Dataset S2*B*), suggesting that, in general, sequence-unrelated effectors may interact with shared target proteins by means of convergent evolution. In other pathosystems, convergence of unrelated effectors from one pathogen onto common host proteins was previously described (14). Using random sampling from an estimated 10,000-protein search space in the cDNA library, we demonstrate that the convergence of different PiRxLRs effectors on common host proteins is highly significant (*P* < 0.0001, empirical test) (*SI Appendix*, Fig. S5). Importantly, it was also shown previously that the extent of convergence correlates with the ability to observe immune phenotypes in *Arabidopsis* genetic knockout lines (14), suggesting that the redundantly targeted potato proteins are likely important for infection. In addition to this convergence, we also noted that effectors from the same family tended to interact with different host proteins, potentially highlighting divergent evolution to acquire new targets (Dataset S2*C*). For example, Pi07387 and Pi22926, which both belong to RxLRfam52, interact with 18 distinct proteins and show no interactors in common with each other.

### PiRxLR Effectors Are Often Unable to Maintain Target Interactions in the Nonhost *Arabidopsis*.

**Fig. 3.**
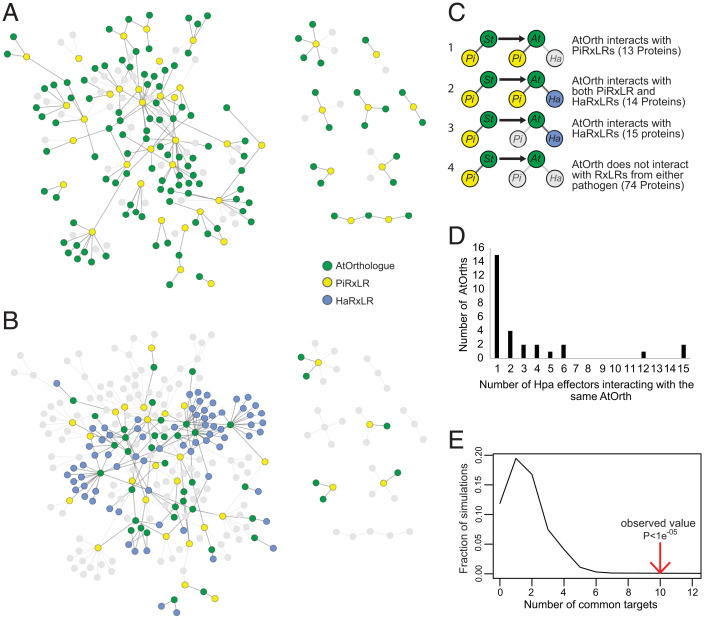
Using a library based on interacting nonhost orthologs enriches for pathogen effector protein–protein interactions (PPIs). (*A*) Network diagram representation of the candidate AtOrths (green circles) of potato cY2H targets, which were identified and cloned. Yellow circles show the PiRxLRs anticipated to interact with cAtOrths based on interactions with potato counterparts (Fig. 2). Straight edges indicate PPIs, and gray circles and edges indicate noncloned orthologs and untestable interactions, respectively. (*B*) Network diagram representing the newly identified MoY2H PPIs detected between cAtOrths (green circles) and HaRxLs (blue circles) or PiRxLRs (yellow circles). AtOrths not cloned or not interacting (gray circles) and untestable or no interactions detected (gray edges) are also shown. (*C*) Schematic representation of the four different categories of PPIs identified alongside an explanation and the numbers involved. Colors and shapes are as described in *B, Arabidopsis thaliana* (*At*)*, Hyaloperonospora arabidopsidis* (*Ha*), *Solanum tuberosum* (*St*). (*D*) Histogram showing the count of AtOrths that interact with a given number of HaRxL effectors. (*E*) The graph shows a significant (*P* < 1e^−05^) increase in HaRxL interactions observed (red arrow) compared with the level expected by random sampling modeling analysis.

To determine whether *P. infestans* effectors maintain interactions with candidate orthologs of their targets in a distantly related nonhost plant, reciprocal best blast hit (RBBH) or best blast hit (BBH) analysis, alongside phylogenetic analyses of orthology available in EnsemblPlants (plants.ensembl.org/index.html), was employed to identify cAtOrths of the putative potato effector targets (Dataset S3). Of 159 cAtOrths, 100 were successfully cloned full-length (FL) de novo, and a further 16 were found to be present in the existing Y2H *Arabidopsis* open reading frame collection (ORFeome) (13, 14), resulting in a matrix candidate AtOrth library (ortholog matrix orthologue yeast two-hybrid [MoY2H]) of 116 *Arabidopsis* proteins. This resulted in a “testable” network of 153 PiRxLR–cAtOrth interactions based on interactions between 40 unique PiRxLRs and 116 unique target candidates. The 116 cAtOrths were screened against the original 64 *P. infestans* effectors (Fig. 3*A*, Dataset S3, and *SI Appendix*, Fig. S6*A*). In addition, they were screened for interaction with 169 HaRxL effectors (Dataset S1*B*). Thirty-four of the HaRxL effectors were represented by more than one allele, cloned from distinct *H. arabidopsidis* genotypes (Dataset S1*B*).

The 116 cAtOrths were cloned into the same Y2H system used in previous matrix Y2H screens (13, 14) to add to those existing screening resources and to facilitate comparisons with those studies. Of the 116 potato cDNAs identified in the cY2H screen (Dataset S2), 42% encoded FL proteins, and a further 18% were missing less than the first 20 amino acids (Dataset S3). Previously, where partial sequences were recovered in the potato cY2H screen, FL sequences that were subsequently cloned retained interactions with corresponding effectors using the Y2H assay (34, 35, 37, 39, 43, 44). To further test this, we selected effector Pi06099, which interacted with a partial StPhyB sequence in the potato cY2H library screen and also interacted with FL AtPhyB in the MoY2H system (Dataset S3). We observed that FL StPhyB and AtPhyB sequences each interacted specifically with Pi06099 in the cY2H system, demonstrating that we can reproduce results in each Y2H system (*SI Appendix*, Fig. S7*A*). We also verified that Pi06099 interacts with both FL StPhyB and AtPhyB in planta using coimmunoprecipitation (coIP) (*SI Appendix*, Fig. S7*B*).

Eighty-nine of the 116 cAtOrths tested did not interact with PiRxLRs; thus, we observed that the majority of *P. infestans* effector–target interactions in potato were apparently not retained in the nonhost *Arabidopsis*, as analyzed using Y2H (126 of 153 interaction pairs that were tested) (Fig. 3*B*). We selected the interaction between Pi21388 (ipi01/AvrBlb1) and CML36, confirming that the effector interacts with FL StCML36 but not with AtCML36 in both the cY2H (*SI Appendix*, Fig. S8*A*) and MoY2H (*SI Appendix*, Fig. S8*B*) systems. Moreover, we confirmed that Pi21388 interacts with StCML36 in planta, but not with AtCML36, using coIP (*SI Appendix*, Fig. S8*C*).

The inclusion of 169 *H. arabidopsidis* effectors in the MoY2H screen resulted in a complex network of interactions (Fig. 3*B*, Dataset S3, and *SI Appendix*, Fig. S6*B*), which could be separated into four broad categories (Fig. 3*C*) based on the interaction status of the AtOrth. In total, 27 of the AtOrths interacted with at least one PiRxLR effector (the original one that interacted with the potato ortholog and/or a different PiRxLR). Of these, 13 AtOrths did not interact with an HaRxL (category 1), while 14 of the AtOrths interacted with effectors from both pathogens (category 2). In contrast, 15 AtOrths did not interact with a PiRxLR but did interact with at least one HaRxL (category 3). There were also 74 AtOrths where no interactions (category 4) were detected, suggesting that these *Arabidopsis* proteins are likely to be significantly sequence divergent to evade the *P. infestans* effector and that, moreover, the *Arabidopsis* protein is perhaps not important for host defense to *H. arabidopsidis* as no equivalent interaction is seen with the HaRxLs tested (Fig. 3*C*).

Strikingly, combining categories 2 and 3 reveals a total of 29 AtOrths that interact with *H. arabidopsidis* effectors (Fig. 3*C*), some of which interact with multiple HaRxLs (Fig. 3*D*). In total, 52 distinct HaRxLs interacted with AtOrth candidate targets (Dataset S3). In some cases where HaRxLs were represented by more than one allele, the alleles interacted with a specific AtOrth. Examples include HaRxL15 alleles that interact with the AtNAC17 transcription factor and the previously reported interactions between HaRxL106 and importin α-isoforms (45). Moreover, six independent ATR1 alleles interact with a mitochondrial small heat-shock protein, perhaps emphasizing this as a strong candidate target for future study (Dataset S3), especially given that it was also an interactor of effectors from *Go* (14). Many of these are candidate targets of *H. arabidopsidis* effectors, which have no known role in defense against *H. arabidopsidis*. Furthermore, the overlap of PiRxLR–potato/AtOrth and HaRxL–AtOrth interactions suggests that perhaps *P. infestans* and *H. arabidopsidis* as oomycete pathogens share common strategies to manipulate plants. We investigated whether the number of interactions between *H. arabidopsidis* effectors and AtOrths was higher than expected by chance, indicating that by selecting orthologs of effector targets from a nonrelated pathogen, we are enriching for host–effector targets/defense components in *Arabidopsis*. Compared with previous unbiased matrix ORFeome Y2H screens to find plant pathogen effector–target interactions (13, 14), the data from our targeted screen show a significant increase in HaRxL interactions (Fig. 3*E*). This suggests that the *P. infestans*–potato cY2H interactome is significantly enriched in potential potato orthologs of *Arabidopsis* proteins that are targets of HaRxL effectors. We performed a gene ontology (GO) term analysis for biological processes of the *Arabidopsis* AtOrths (Dataset S3). Twelve GO terms were enriched among the AtOrths (*SI Appendix*, Fig. S9). Interestingly, these include “Golgi to plasma membrane transport” and “vesicle-mediated transport to plasma membrane,” in agreement with a recent report that *P. infestans* effector targets may be enriched for vesicle trafficking (46). These data indicate that the common host–pathogen interaction interface described in Weßling et al. (14) as being converged on by evolutionarily diverse pathogens may exist in similar form in diverse plant host species.

### Expression of AtOrths in a Susceptible *P. infestans* Host Plant Can Alter Resistance.

**Fig. 4.**
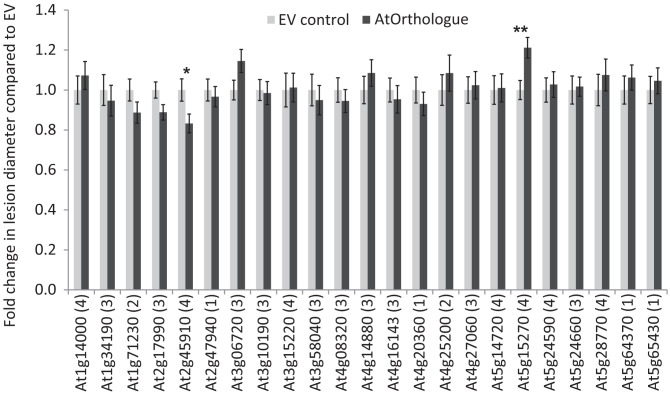
Screening of selected cAtOrths for altered resistance. The graph shows *P. infestans* lesion diameters following *Agrobacterium*-mediated transient expression of cMYC-cAtOrths in *N. benthamiana*. Measurements were taken at 7 days post-infection (dpi), and data for each ortholog are expressed as a fold change to the internal cMYC empty vector (EV) control, which was normalized to a value of one. Error bars are SE. The graph shows combined data from greater than or equal to three independent replications of each experiment (*n* ≥ 108). Numbers in parentheses represent the interaction category (Dataset S3). Asterisks indicate significant differences as tested pairwise by the Student’s *t* test or the Mann–Whitney rank sum test. *P ≤ 0.05; **P ≤ 0.01.

The *P. infestans* and *H. arabidopsidis* effectors tested performed poorly in the nonhost system (Fig. 1), and in the majority of cases, the *P. infestans* effectors were unable to maintain their interactions with cAtOrth (Fig. 3*B* and Dataset S3). Hence, we hypothesized that if the AtOrth did not interact with or could not be correctly manipulated by the corresponding *P. infestans* RxLR effector, the AtOrth could compensate for the loss of the native host protein being targeted by the effector, provided it retained its function. To test this, a selection of AtOrths was transiently expressed in *N. benthamiana* to assess whether they could alter susceptibility to *P. infestans* in a host plant. Protein expression for each AtOrth is shown in *SI Appendix*, Fig. S10. The AtOrths were selected to represent a range of interaction categories (Table 1). Of the 23 AtOrths selected for expression in *N. benthamiana*, 17 represented categories 3 and 4 (i.e., no interaction of the *P. infestans* effector with the *Arabidopsis* protein). Only expression of AT5G15270 and AT2G45910, both category 4 interactors, significantly altered *P. infestans* infection levels (Fig. 4). AT5G15270 overexpression enhanced *P. infestans* colonization compared with the control. Interestingly the potato equivalent, StKRBP1, acts as an S factor, and its overexpression also boosts *P. infestans* colonization of *N. benthamiana* (33). Although At5g15270 does not interact with Pi04089 in either the cY2H (*SI Appendix*, Fig. S11*A*) or MoY2H (*SI Appendix*, Fig. S11*B*) systems and did not interact in planta using coIP (*SI Appendix*, Fig. S11*C*), StKRBP1 is able to homodimerize and also, to weakly coimmunoprecipitate At5g15270 (*SI Appendix*, Fig. S11*D*). Moreover, StKRBP1 and At5g15270 colocalize at nuclear speckles (*SI Appendix*, Fig. S11*E*). This indicates that the candidate orthologs may form part of the same complex in planta. In contrast, transient overexpression of AT2G45910 (AtPUB33) resulted in a significant decrease in *P. infestans* colonization (Fig. 4), and this gene was thus studied in greater detail.

**Table 1.** cAtOrths selected for expression in *N. benthamiana*

cAtOrths	*Arabidopsis* annotation	PiRXLR interacting in potato cY2H	Interacts in MoY2H with PiRxLRs	Interacts in MoY2H with HpaRxLRs	Interaction category of cAtOrth
AT1G14000	AtVIK, VH1-interacting kinase	Pi17309, Pi17316	No	No	4
AT1G34190	NAC domain containing protein 17	Pi03192	No	Yes	3
AT1G71230	Encodes a subunit of the COP9 complex	Pi07555, Pi13625, Pi13959	Yes	Yes	2
AT2G17990	AtCAP2, Ca-dependent protein kinase adaptor	Pi15287, Pi04339, Pi07387	No	Yes	3
AT2G45910	AtPUB33, UBOX and kinase domain protein	Pi06087	No	No	4
AT2G47940	AtDegP2 protease	Pi10654	Yes	No	1
AT3G06720	AtIMPA-1 importin-α	Pi22798	No	Yes	3
AT3G10190	AtCML36, calmodulin-like 36	Pi21388	No	Yes	3
AT3G15220	Protein kinase superfamily protein	Pi13628	No	No	4
AT3G58040	Encodes an RING finger domain protein	Pi04339	No	Yes	3
AT4G08320	AtTPR8, tetratricopeptide repeat 8	Pi07689, Pi14371	No	Yes	3
AT4G14880	*O*-acetylserine(thiol)lyase	Pi14371	No	Yes	3
AT4G16143	AtIMPA-2, importin α-isoform 2	Pi22798	No	Yes	3
AT4G20360	AtSVR11, suppressor of variegation 11	Pi22926	Yes	No	1
AT4G25200	AtHSP23.6-MITO, small heat-shock protein 23	Pi07689	Yes	Yes	2
AT4G27060	AtTORTIFOLIA1, microtubule-associated protein	Pi06308	No	Yes	3
AT5G14720	Protein kinase superfamily protein	Pi11383	No	No	4
AT5G15270	RNA binding KH domain–containing protein	Pi04089	No	No	4
AT5G24590	AtTIP, TCV-interacting protein, AtNAC91	Pi09218	No	No	4
AT5G24660	AtLSU2, response to low sulfur 2	Pi15287	No	Yes	3
AT5G28770	bZIP protein BZO2H3	Pi07555	No	No	4
AT5G64370	AtPYD3 encodes a β-ureidopropionase	Pi15278	Yes	No	1
AT5G65430	ATMIN10, general regulatory factor 8	Pi02860	Yes	No	1

### Expression of AtPUB33 Reduces *P. infestans* Colonization of Host Plants.

**Fig. 5.**
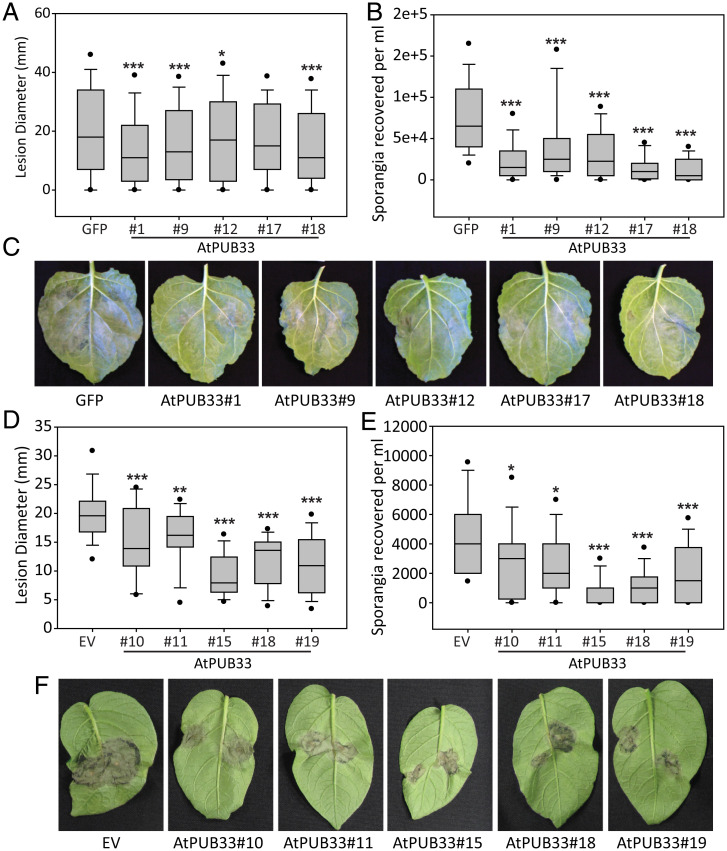
Transgenic plants overexpressing AtPub33 show increased resistance to *P.infestans*. (*A*) The box plot shows *P. infestans* lesion diameters in five independent transgene generation 2 (T_2_) *N. benthamiana* lines expressing cMYC-AtPUB33 compared with a T_2_ cMYC-GFP control. (*B*) The box plot shows *P. infestans* sporangia recovered per milliliter in five independent T_2_ *N. benthamiana* lines expressing cMYC-AtPUB33 compared with a T_2_ cMYC-GFP control. (*C*) Representative leaf images showing *P. infestans* lesions on five independent T_2_ *N. benthamiana* lines expressing cMYC-AtPUB33 compared with a T_2_ cMYC-GFP control. (*D*) The box plot shows *P. infestans* lesion diameters in five independent potato transgenic lines expressing untagged AtPUB33 compared with an empty vector (EV) control. (*E*) The box plot shows *P. infestans* sporangia recovered per milliliter in five independent potato transgenic lines expressing untagged AtPUB33 compared with an EV control. (*F*) Representative leaf images showing *P. infestans* lesions on five independent potato transgenic lines expressing untagged AtPUB33 compared with an EV control. Graphs and box plots show combined data from greater than or equal to three independent replications of the experiments. Circles on box plots indicate 5th and 95th percentile outliers. Asterisks indicate significant differences as tested by one-way ANOVA or Kruskal–Wallis one-way ANOVA on ranks with multiple comparisons vs. the control group using the Holm–Sidak method. *P ≤ 0.05; **P ≤ 0.01; ***P ≤ 0.001.

AtPUB33 is an E3 ubiquitin ligase but is unique within the class of plant U-box (PUB) domain–containing proteins as it also contains a kinase domain. This study and previous work have shown that the predicted potato ortholog of AtPUB33, called StUBK (PGSC0003DMT400000146), is targeted by the *P. infestans* effector Pi06087 (PiSFI3/PexRD16) (34). As AtPUB33 belonged to category 4 (no interactors from either pathogen) in the MoY2H screen, Y2H pairwise tests were carried out confirming that AtPUB33 does not interact with Pi06087, whereas StUBK does (*SI Appendix*, Fig. S12). To verify the data from the transient assay, stable transgenic lines overexpressing AtPUB33 were constructed in both *P. infestans* host species: cMYC-AtPUB33 in *N. benthamiana* and untagged AtPUB33 expression in potato (*SI Appendix*, Fig. S13). Five independent transgenic lines were selected for each plant species where there was production of detectable myc-PUB33 protein (*SI Appendix*, Fig. S13 *A* and *B*) or detectable *AtPUB33* transcript (*SI Appendix*, Fig. S13*C*). The transgenic potato and *N. benthamiana* plants were subsequently challenged with *P. infestans* alone and found to have significantly lower pathogen colonization (Fig. 5 *A* and *D*) as measured by smaller lesion sizes (Fig. 5 *B* and *E*) and fewer sporangia recovered from the leaf surface (Fig. 5 *C* and *F*) compared with the cMYC-GFP or empty vector controls, respectively. This confirms the reduction in plant susceptibility to *P. infestans* observed transiently (Fig. 4). We propose that the reduction in susceptibility is due to AtPUB33 not being targeted by the effector and complementing loss of StUBK activity (the potato protein targeted by Pi06087), hence overcoming the impact of the effector. It is possible that the reduction in susceptibility could have been due to overexpression of AtPUB33 per se, and indeed, we did not generate transgenic lines overexpressing StUBK for direct comparison. However, we used transient expression experiments to demonstrate that AtPUB33 specifically undermines Pi06087 effector function. Transient expression of Pi06087 in transgenic *N. benthamiana* plants expressing GFP led to enhanced *P. infestans* colonization. This increase in pathogen colonization was not observed on plants expressing AtPUB33 (*SI Appendix*, Fig. S14). In contrast, expression of control effector Pi04089 enhanced *P. infestans* colonization on both GFP- and AtPUB33-expressing plants (*SI Appendix*, Fig. S14). These data indicate that expression of the cAtOrth AtPUB33 in host plants specifically undermines the virulence function of the effector, Pi06087, consistent with the effector failing to interact with and thus, manipulate AtPUB33. This provides support for the hypothesis that plant immunity can be enhanced through overexpression of nonhost orthologous proteins that escape effector manipulation.

## Discussion

### Many Effectors Fail to Enhance Susceptibility in Nonhost Plants.

We selected RxLR effectors that enhance susceptibility when expressed in host plants—15 *P. infestans* effectors (28) and 10 *H. arabidopsidis* effectors (ref. 19 and this work)—and tested whether they would also enhance susceptibility in nonhost plants. Only three *H. arabidopsidis* effectors enhanced *P. infestans* colonization in *N. benthamiana*, whereas no *P. infestans* effectors enhanced *H. arabidopsidis* colonization in *Arabidopsis* (Fig. 1). Likely explanations for these failures are 1) that the effectors are unable to suppress immunity in the nonhost plants or 2) that the requirements for susceptibility differ for these two oomycete pathogens, one of which is a hemibiotroph and the other of which is an obligate biotroph. A third explanation, that the effectors are recognized by resistance proteins and, thus, trigger ETI, is unlikely as their expression did not trigger cell death in the nonhost plants.

A previous study demonstrated that the very few RxLR effectors conserved at the protein sequence level between *H. arabidopsidis* and *Phytophthora* species can suppress PTI and ETI in distantly related nonhost plants (30, 31). However, most *H. arabidopsidis* effectors are not conserved with *Phytophthora* species (7), and that was the case for those tested in Fig. 1. Both hypotheses to explain the failures of most of these effectors to enhance colonization in the nonhost plants are consistent with either the independent evolution of effectors with different roles in the two pathogens or the significant divergence of effectors from a common starting point. Nevertheless, three HaRxLs did enhance *P. infestans* colonization of *N. benthamiana*, suggesting that they function in this nonhost plant, albeit they were significantly less proficient at doing so compared with most PiRxLRs tested. This is perhaps indicative of effectors that only poorly interact with or are less efficient in appropriately manipulating orthologs of their targets in the nonhost. The effector HaRxL21, which significantly enhances *P. infestans* colonization of *N. benthamiana* (Fig. 1), targets the transcriptional repressor TOPLESS in the host *Arabidopsis* (47), raising the possibility that it is also capable of targeting this host protein in *N. benthamiana*. Interestingly, effector HaRxL44 interacts with the *Arabidopsis* mediator subunit med19a (20), which was confirmed here (Dataset S3), but failed to enhance *P. infestans* colonization, raising the possibility that it also fails to appropriately manipulate med19a in *N. benthamiana*. In contrast to the *H. arabidopsidis* effectors, no *P. infestans* effectors enhanced *H. arabidopsidis* colonization of *Arabidopsis* across all transgenic lines tested. Indeed, expression of two effectors, Pi04089 and Pi10654, consistently reduced colonization by *H. arabidopsidis*. Whether this is due to triggering resistance or failing to provide a metabolic change required for susceptibility remains to be tested. Interestingly, however, Pi04089 accelerated flowering time (*SI Appendix*, Fig. S3), perhaps suggesting 1) that it does indeed target an *Arabidopsis* protein but that this is an off-target interaction; 2) that it fails to appropriately manipulate its target; 3) that *Arabidopsis* and potato differ in their regulatory networks controlling immunity; or 4) that *H. arabidopsidis* and *P. infestans* have different requirements for susceptibility. In contrast to the results here, most tested candidate effectors from the poplar rust fungus *Melampsora larici-populina* enhanced *H. arabidopsidis* colonization when expressed in *Arabidopsis* (48). This suggests that these *M. larici-populina* effectors are able to interact with and appropriately manipulate their targets in *Arabidopsis*. In the case of *M. larici-populina*, the pathogen lifestyle is very similar to *H. arabidopsidis*, in that both are obligate biotrophs, so defense responses to each may also be similar.

### *P. infestans* RxLRs Interact with Diverse Host Proteins and Target Hubs Shared with Other Pathogens.

Sixty-four previously described PiRxLRs (Dataset S1) (28) were screened in a cY2H library derived from RNA extracted from potato–*P. infestans* interactions (32). Twenty-four effectors revealed no interacting proteins, potentially indicating that their targets are not proteins; are proteins associated with host membranes and, thus, interactions cannot be demonstrated in Y2H; or are proteins, or regions of proteins, that are not represented in the cY2H library (Fig. 2 and Dataset S2). The remaining 40 PiRxLRs interacted with 169 host proteins in the cY2H screens. Some host proteins were represented by multiple yeast clones emerging from a screen. All such interactions that have been examined in more detail have been verified in planta using coIP and/or bimolecular fluorescence complementation (32–44). Moreover, interactions between effector Pi06099 and StPhyB and between effector Pi21388 and StCML36 were also verified in planta here (*SI Appendix*, Figs. S7 and S8). The cY2H screens thus offer many high-confidence candidate target proteins and processes that are potentially manipulated by *P. infestans* effectors in its host potato during infection, and thus, they provide a valuable resource to the research community.

The candidate targets of the 40 PiRxLRs represent proteins involved in several biochemical processes, including phosphorylation or dephosphorylation, ubiquitination, DNA or RNA binding, lipid binding, protein binding, and various enzymatic activities (Dataset S2). A range of highly diverse host proteins and processes are thus implicated as targets for manipulation during late blight disease. Very few candidate membrane receptor proteins associated with signal perception were observed, potentially because interactions with such proteins would be unlikely to occur in Y2H. Interestingly, not many candidate targets were revealed that are associated in previous studies with immunity. Those that were included signal transduction components, such as the mitogen-activated protein (MAP) kinase kinase kinases MAP3Kβ2 (44), MAP3Kɛ (37), and MAP3K5 (49) and transcriptional regulators, such as MYC2 (50) and Med19a (20).

Analysis of GO terms for biological processes among the AtOrth candidates (Dataset S3) revealed enrichment of 12 processes (*SI Appendix*, Fig. S9), including Golgi to plasma membrane transport and vesicle-mediated transport to plasma membrane. A recent report of proteomic data generated following immunoprecipitation of *P. infestans* effectors expressed in *N. benthamiana* (46) revealed enrichment of secreted proteins and proteins involved in vesicle trafficking. Our observations agree that secretion and subcellular trafficking are likely RXLR effector targets in the host.

Most *P. infestans* effectors (48) interact with more than 2 host proteins, with the maximum number being 18. In detailed studies, it has been shown that effectors, such as Avr-Piz-t from *Magnaporthe oryzae*, interact with functionally diverse proteins, such as the plasma membrane potassium channel OsAKT1 (51), the ubiquitin E3 ligases APIP6 (52) and APIP10 (53), the basic leucine zipper (bZIP) transcription factor APIP5 (54), and the nucleoporin-like protein APIP12 (55), suggesting multiple functions. Indeed, Avr3a from *P. infestans* has been reported to interact with the ubiquitin E3 ligase CMPG1 (32) and to associate in planta with dynamin-related protein 2 (56) and AtCAD7 (57). Multifunctional effectors are also a feature of bacterial type III secretion system arsenals (15), indicating that effectors can potentially play multiple virulence roles. In addition, we saw intraspecies convergence of multiple effectors on common host proteins. This was previously described by Weßling et al. (14), where it was shown that the extent of convergence correlates with the frequency of immune phenotypes for the respective genetic null mutants. Thus, while it is possible that some of these are false-positive interactions, the interaction topology has been observed before and is likely related to the biology of host–microbe interactions.

It is interesting to note that some candidate targets of PiRxLR effectors in potato are common targets of other pathogens, some of which are also regarded as highly interconnected regulatory hubs, such as CSN5 and PRA1. Other common targets include the endoplasmatic reticulum associated transcription factor NTL9, LSU2, SEC5, importin-α, kinesin, SINA2, HSP23, Ftn2, Med19a, and 14-3-3 proteins (Dataset S4) (13, 14). While this interspecies convergence was described by Weßling et al. (14), our data suggest that similar host proteins are relevant for related pathogens in different plant species and hence, that the molecular pathogen–host interface is similar not only for different pathogens targeting the same host but also, for related pathogens targeting different hosts.

### The Candidate Ortholog Y2H Screen.

In the MoY2H screen, most PiRxLRs failed to interact with the cAtOrths of their potato cY2H interactors (Fig. 3 and Dataset S3). This suggests that in many cases, sequences of the putative orthologs had diverged sufficiently to abolish recognition due to the evolutionary distance between potato and *Arabidopsis*. Although the two Y2H screens differ in that the MoY2H system comprised FL candidate AtOrth sequences, whereas the potato cY2H library yielded a mixture of FL and truncated cDNAs, ∼60% of potato cDNAs emerging from the cY2H screen were FL or almost so (Dataset S3), and we have observed that cloning FL versions of truncated sequences emerging from the cY2H screen produces similar interaction results (*SI Appendix*, Figs. S11 and S13) (34, 37, 39, 40, 43, 44). Therefore, failure of so many effectors to interact with cAtOrths may help to explain why *P. infestans* is unable to colonize *Arabidopsis* and provides support for the model proposed by Schulze-Lefert and Panstruga (5), which proposes that many effectors may fail to manipulate their targets in distant nonhost plants presumably due to sequence divergence, at least in regions of interaction with the effectors.

The four distinct interaction categories of cAtOrths uncovered in the ortholog screen suggest several possibilities. Category 1 cAtOrths, where PiRxLR effectors interact but there were no interactions with *H. arabidopsidis* effectors, suggests that although targets are sufficiently conserved at the protein level, they may not play a role in regulating immunity to *H. arabidopsidis* or effectors from *H. arabidopsidis* may target alternative proteins to alter the same host processes. Category 2 cAtOrths, which interacted with effectors from both pathogens, could indicate that there is conservation both of protein structure and of infection strategies employed by both pathogens. Category 3 cAtOrths, where PiRxLRs failed to interact but *H. arabidopsidis* effectors did, also support the common infection strategies hypothesis. In fact, categories 2 and 3 revealed that a disproportionately large percentage of the cAtOrths interacts with *H. arabidopsidis* RxLRs, which is statistically significant when compared with a random selection of *Arabidopsis* proteins. Indeed, this screen has identified many proteins that interact with *H. arabidopsidis* effectors with no previously known roles in immunity. In contrast, the large number of cAtOrths belonging to category 4, where there was no interaction with effectors from either pathogen, underlines the evolutionary distance between the two pathosystems and may suggest either that the regulatory systems controlling immunity differ between the two plants or that the pathogens also employ different strategies for immune suppression that better suit their different infection cycles. For example, *H. arabidopsidis* is an obligate biotroph that relies on keeping its host alive to complete its life cycle. In contrast, hemibiotrophic pathogens, such as *P. infestans*, maintain living host cells for a shorter period before switching to a necrotrophic phase in which host cells may be actively killed. However, infection by the obligate biotroph *Albugo* allows colonization of *Arabidopsis* by *P. infestans*, thus breaking NHR, whereas *H. arabidopsidis* does not allow this (25). Interestingly, *Albugo* employs a different infection strategy to *H. arabidopsidis* in that it is more tolerant of certain *Arabidopsis* immune responses and better able to colonize these plants under stress conditions (58).

None of the *Arabidopsis* lines expressing *P. infestans* RxLRs consistently enhanced *H. arabidopsidis* infection to statistically significant levels (Fig. 1) whether they maintained interactions with candidate cAtOrths (Pi02860, Pi04314, Pi06099, Pi07387, Pi07555, Pi10654, Pi22798, and Pi22922) or failed to interact with them (Pi04089, Pi06087, Pi09218, Pi09585, Pi13628, Pi17316, and Pi22926) (Dataset S3). Potato interactors of Pi02860 (StNRL1) and Pi04314 (PP1c isoforms) are verified targets of these effectors (39, 40). Failure of these effector transgenic lines to enhance *H. arabidopsidis* colonization may suggest that these targets are inappropriately manipulated in *Arabidopsis* or that their manipulation is not productive for *H. arabidopsidis* infection. In contrast, failure of Pi06087 (SFI3), Pi09858 (SFI4), and Pi13628 (SFI5) to interact with cAtOrths is consistent with the previously reported inability of these effectors to suppress PTI in *Arabidopsis* (59).

Of course, not all cAtOrths were cloned and tested, and many effectors interacted with multiple targets, where some interactions were maintained but others were not. However, there are examples where PiRxLR expression significantly reduced *H. arabidopsidis* colonization of *Arabidopsis* (Pi04089 and Pi10654), suggesting that these effectors exhibit an activity whether they interact with the cAtOrth of their host potato interactor or not. Pi04089 is interesting as the transgenic lines also had an early flowering phenotype, and although Pi04089 did not interact with At5g15270 (*SI Appendix*, Fig. S11), several closely related family members of this RNA binding protein in *Arabidopsis* are known to regulate flowering time (60), suggesting an off-target action of the effector. In this regard, it is interesting to note that whereas KRBP1 acts as a susceptibility factor (33), its closely related homolog FLK is a positive regulator of flowering (61), and a recent preliminary report suggests that it is also a positive regulator of plant immunity (62). It will be interesting to see whether Pi04089 interacts with a closely related K homology (KH) RNA binding protein, such as FLK. In contrast, whereas the interaction is maintained between Pi10654 and AtDegP, failure of transgenic lines expressing this effector to enhance pathogen colonization suggests that the effector is not able to correctly manipulate the cAtOrth , that manipulation differentially regulates immunity between *Arabidopsis* and potato, or that the *H. arabidopsidis* infection process does not have the same requirements as that of *P. infestans*. Indeed, there are many examples where mutation of effector targets can have both an enhanced disease resistance or enhanced disease susceptibility phenotypes in response to challenge with different pathogens or even different strains of the same pathogen (14).

### Expression of Nonhost Targets in Host Plants.

Historically, engineering disease resistance has often involved transfer of *R* genes from one plant species to another (63, 64). Transfers typically make a huge impact on defense by enhancing the recognition specificity but can be overcome by rapidly evolving pathogens through mutation or loss of the recognized effector. Pyramiding multiple *R* genes is expected to enhance the durability of such resistance. Increases of recognition specificity can also be engineered through the transfer of pathogen-associated molecular pattern receptors, such as the EF-Tu (elongation factor thermo unstable) receptor to new species to help combat disease (65). Other approaches to enhance immunity involve the mutagenesis or knockdown of so-called *S* genes, which are required by the pathogen for a successful colonization of its host (63, 66, 67). Here, we provide proof of concept for an approach to enhance disease resistance by transfer of effector target proteins from nonhost to host plants. Expression of AtPUB33, a nonhost *Arabidopsis* ortholog of the effector target StUBK (34), increased resistance to *P. infestans* in two different host species, potato and *N. benthamiana* (Fig. 5). As AtPUB33 failed to interact with the PiRxLR effector Pi06087/SFI3 (*SI Appendix*, Fig. S12), it presumably is not targeted by *P. infestans* for manipulation by this effector. Pi06087 did not enhance pathogen colonization on transgenic plants expressing AtPUB33 (*SI Appendix*, Fig. S14). This result indicates that although effector Pi06087 can target potato StUBK and presumably prevent its function, it is unable to target AtPUB33, leaving the nonhost ortholog able to effectively complement for the effector-mediated loss of StUBK activity. Although the level of enhanced resistance was modest compared with *R* gene introgression, pyramiding could incorporate multiple nonhost genes with additive effects on disease resistance through escaping pathogen effector manipulation. It may be possible to use RNA editing/CRISPR to mutate discreet effector-interacting regions of host effector target proteins to resemble the nonhost variant. Identifying nonhost effector target orthologs that evade manipulation could provide a strategy to promote durable disease resistance.

## Materials and Methods

### Plant Growth.

*N. benthamiana* was grown at 22 °C in 16-h days and 8-h nights at 18 °C. Ambient light was maintained between 200 and 450 W/m^2^. *A. thaliana* was grown at 20 °C with 12-h day length.

### Effector Cloning.

*H. arabidopsidis* RxLR candidates were amplified from cDNA from spores and infection. The *P. infestans* effector collection was generated as described (28). *H. arabidopsidis* and *P. infestans* candidate effectors were cloned minus the signal peptide (Dataset S1). Dataset S1 shows primer sequences for the addition of attachment site B (ATTB) recombination sites by nested PCR. Recombination of attB–effector PCR products with pDonrZeo or pDonr201 was performed to generate Gateway entry clones. Effectors were recombined into pB7WGF2 (68) or pEG201 (69) destination vectors and transferred into *Agrobacterium* to conduct transient assays or make transgenic *Arabidopsis*.

### Generation of *Arabidopsis* Transgenics.

*Arabidopsis* ecotype Col-4 was dipped (70) with *Agrobacterium* harboring PiRxLRs cloned into pEG201 (69) expressed with a Cauliflower mosaic virus (CaMV 35S) promoter. Lines were selected on Basta soaked soil (1 mL/L) until homozygosity at T3. Three independent lines were generated for each effector. Expression of *P. infestans* effector messenger ribonucleic acid (mRNA) in *Arabidopsis* transgenics was verified using RT-PCR. RNA was extracted from pooled 14-d-old seedlings using a RNeasy Plant Mini Kit according to the manufacturer but with the addition of deoxyribonuclease (DNase) treatment using a Qiagen RNase-Free DNase Set. cDNA was synthesized using SuperScript II reverse transcriptase. PCR was performed using Bioline Biomix Red. Dataset S6 shows primer sequences.

### *H. arabidopsidis* Infection Assays.

Infections with *H. arabidopsidis* isolate Noks1 were performed on 2-wk-old seedlings as described (71). Noks1 was maintained on 7-d-old *Arabidopsis* ecotype Col-0 seedlings. Spores were harvested from infected Col-0 seedlings, filtered through miracloth, and adjusted to 30,000 spores/mL. Sporangiophores per seedling were counted 4 days post-infection (dpi) using a dissecting microscope (15 plants per pot, three pots per tray, two replicates with at least two lines per transgenic).

### *P. infestans* Culture snd Infection Assays.

Sporangia were prepared from *P. infestans* strain 88069 after growth at 19 °C on Rye agar plates for 11 to 14 d. Sporangia were harvested (72) to a concentration of 50,000/mL in sterile distilled water (SDW). Leaves (three per plant; greater than or equal to six plants per replicate; less than or equal to three replicates) of transgenic *N. benthamiana* and potato lines (three leaves per plant; four plants per replicate; two replicates.) were drop inoculated with 10 μL of *P. infestans* inoculum. Lesions were measured at 7 dpi. Sporangia were harvested from leaves in 3 mL of sterile water and counted using a counting chamber.

### *Agrobacterium*-Mediated Transient Infection Assays.

*Agrobacterium* strains GV3101 or AGL1 expressing PiRxLR or cAtOrth constructs were grown at 28 °C overnight in yeast extract and beef media supplemented with appropriate antibiotics. Cultures were pelleted at 4,000 rpm before resuspension in 10 mM MES (2-(N-morpholino)ethanesulfonic acid): 10 mM MgCl_2_ with 200 μM acetosyringone adjusted to an optical density (OD) at 600nm of 0.1. *Agrobacterium* control and test samples were infiltrated on either half of an *N. benthamiana* leaf (three per plant; greater than or equal to six plants per replicate; greater than or equal to three experimental replicates) before being drop inoculated 24 h later with 10 μL *P. infestans* inoculum (50,000 sporangia/mL). Infection lesions were measured at 7 dpi, and Student’s *t* tests or Mann–Whitney rank sum tests were performed to determine statistical significance.

### Potato Y2H Screens.

Screens were conducted with the Invitrogen ProQuest system and yeast strain MaV203 according to the manufacturer. DNA binding domain “bait” fusions to each *P. infestans* effector were generated using Gateway recombinations from relevant pDonr201 clones. These were transformed into MaV203 cells and recovered by nutritional selection, and subsequently, they were tested to eliminate reporter gene autoactivation. Competent cells were generated for each bait construct and transformed individually with a potato cDNA activation domain “prey” cY2H library. Interacting clones were selected as described previously (72). Interacting clones were sequenced to determine the interacting plant protein, and clones were cotransformed into yeast to confirm interaction and tested for prey autoactivation.

### Ortholog Identification and Cloning.

cAtOrths of potato Y2H interactors were found by performing RBBH analysis between the two genomes. If no RBBH was found, the BBH was taken. In addition, we used the EnsemblPlants (plants.ensembl.org/info/genome/compara/homology_method.html) phylogenetic study of candidate orthologs as an independent assessment. The coding sequences of the cAtOrths were found to be present in the existing matrix Y2H *Arabidopsis* clone library (13, 14) or amplified by nested PCR to add ATTB recombination sites, and they were recombined into Gateway entry vectors. cAtOrth sequences and primers are shown in Dataset S5.

### Convergence Analysis.

We conservatively model that 10,000 different proteins are represented in the potato cY2H library. To estimate the significance of convergence, we randomly sampled (*n* = 215) interactions with 10,000 available proteins (random sampling with replacement). In each iteration, the number of distinctly targeted proteins was counted, and the random density distribution was plotted and compared with the number of experimentally observed distinct targets (*n* = 169). To ensure robustness, we repeated the analysis with smaller search spaces of 5,000 and 2,000 proteins, resulting in the same conclusion.

### *Arabidopsis* Candidate MoY2H Screens.

The Y2H assay was performed as described (12) with minor modifications. Open reading frames (ORFs) coding for *H. arabidopsidis* and *P. infestans* effector candidates were transferred into DNA-binding domain (DB) containing pDest-DB vectors, and recombinants were confirmed by PCR and Sanger sequencing. Isolated pDest-DB clones were transformed into *Saccharomyces cerevisiae* Y8930 (mating type α) by lithium–acetate transformation. Positive transformants were selected on medium lacking leucine, and archival stocks were prepared and stored at −80 °C. cAtOrths were cloned by Gateway recombinant cloning in pDEST-AD and verified by PCR and Sanger sequencing (Dataset S5). pDEST-AD clones containing cAtOrths were transformed in *S. cerevisiae* Y8800 (mating type a) by lithium–acetate transformation, and positive transformants were selected and stored in 40% glycerol at −80 °C. Autoactivator removal was performed as described (14). For the primary Y2H screen, pDest-AD-Ortholog clones were grown on synthetic complete solid medium lacking tryptophan for 2 d, and pools of 75 individuals were generated. Uniform distribution of clones was checked as described (12). Single DB–effector clones were mated with pools of 75 AD-cAtOrth clones. Five microliters of freshly grown DB and AD yeast was spotted on top of each other on yeast extract peptone dextrose growth medium (YEPD) using a liquid handling robot. Identification of interacting effector–ortholog clones was as described (12, 13, 73). The screen was repeated once. Interactions were verified when they were positive in three of four repeated matings and autoactivation was not detected. Methods to define effector ortholog–protein interactions were as described (13, 73). Consequently, key parameters of the interactome screen, such as sampling and assay sensitivity, are identical between experiments, and integration of data will not introduce bias due to the experimental design (74).

### Common Targets between *H. arabidopsidis* and *P. infestans* Effectors.

To assess whether *H. arabidopsidis* and *P. infestans* effectors have more common *Arabidopsis* targets than expected, the number of distinct targets in both screens was compared with 10,000 random picks from the Arabidopsis Interactome version 1 “main screen” (AI-1 MAIN) (73), previously used for effector–host interaction screening. To model the expectation of finding *H. arabidopsidis* effectors by unbiased screening, we randomly picked 10,000 times 116 target proteins (i.e., the number of *P. infestans*–potato target orthologs that we tested for interactions with *H. arabidopsidis* effectors) and counted the number of real *H. arabidopsidis* effector targets observed previously (13, 73). Comparing the observed value of 10 *H. arabidopsidis* effector interactions with *P. infestans* effector target orthologs with random distribution shows a significantly higher rate of interaction detection using the ortholog approach than using unbiased screening.

### Generation of *N. benthamiana* Transgenics.

Approximately 40 small leaf disks per construct of *N. benthamiana* leaves agroinfiltrated with CaMV 35S-driven expression of myc-GFP or myc-AtPUB33 (OD600 = 0.05) were harvested at 2 dpi and surface sterilized in 2% bleach with 1 drop of Tween20 per 50 mL for 10 min. Leaf disks were washed five times in SDW and aseptically transferred to shoot-inducing media plates (Murashige and Skoog medium (MS), 2% sucrose, 0.8% agar, 2 mg/L 6-benzylaminopurine (BAP), 0.5 mg/L 1-Naphthaleneacetic acid (NAA), 200 μg/mL timentin, 50 μg/mL kanamycin). Plates were renewed every 10 d for ∼2 to 3 mo until shoots appeared. Shoots were then transferred to root-inducing media (MS, 2% sucrose, 0.8% agar, 0.5 mg/L NAA, 200 μg/mL timentin, 50 μg/mL kanamycin). On rooting, plantlets were transferred to soil. Positive transformants were confirmed by immunoblot; five individual lines per construct with detectable protein expression were recovered. Seeds collected from T0 and T1 plants were sown on MS supplemented with kanamycin selection, and transgene expression was confirmed by immunoblots.

### Generation of Potato Transgenics.

Transgenic potatoes expressing an untagged form of AtPUB33 under a CaMV 35S promoter and nopaline synthase (Nos) terminator were made by Simplot Plant Sciences (J. R. Simplot Company) as described (75), except that kanamycin was used as a selectable marker.

### Immunoblotting.

Transgenic *N. benthamiana* plant lines or protein fusions transiently overexpressed at 2 dpi in *N. benthamiana* were tested by immunoblotting to assess protein presence and stability. Proteins were extracted using GTEN extraction buffer (10% glycerol; 25 mM Tris, pH 7.5; 1 mM Ethylenediaminetetraacetic acid (EDTA); 150 mM NaCl; 1 mM phenylmethylsulfonyl fluoride (PMSF); 10 mM dithiothreitol (DTT); 0.5% Nonidet p40; protease inhibitor tablet) mixed with 2× sodium dodecylsulfate polyacrylamide gel electrophoresis (SDS-PAGE) sample buffer, loaded onto 12% SDS-PAGE gels, and run for 2 h at 120 V. Gels were blotted with nitrocellulose membrane for 1.5 h at 30 V with Ponceau staining to demonstrate transfer and loading. Membranes were blocked in 5% milk in 1× PBST (137 mM NaCl; 12 mM phosphate; 2.7 mM KCl, pH 7.4; 0.2% Tween20) for 1 to 2 h before the addition of primary antibodies overnight: a monoclonal GFP antibody raised in mouse at 1:2,000 dilution (sc9996; Santa Cruz), a monoclonal anti-cMYC antibody raised in mouse at 1:500 (catalog no. SC-40; Santa Cruz), a monoclonal red fluorescent protein (RFP) antibody raised in rat at 1:4,000 (catalog no. 5F8; Chromotek), or a monoclonal anti-HA antibody produced in rat at 1:1,000 (3F10; Sigma). The membrane was washed with 1× PBST (0.2% Tween20) five times for 5 min each before the addition of secondary antibody at 1:5,000 dilution with either anti-mouse immunoglobulin horseradish peroxidase (Ig-HRP) antibody (A9044; Sigma-Aldrich) or anti-rat Ig-HRP (ab6836; Abcam) for 1 h at room temperature followed by five more washes and enhanced chemiluminescence (ECL) (Amersham) development according to the manufacturer.

### coIP.

Protein fusion constructs were transiently overexpressed in *N. benthamiana* using *Agrobacterium*-mediated expression. Leaf disks were collected 48 h after infiltration. Samples were ground in liquid nitrogen, and tissue was resuspended in 500 µL GTEN extraction buffer (as above), gently vortexed, placed on ice for 10 min, and centrifuged at 13,000 × *g* for 10 min at 4 °C. For input samples, 40 µL of sample was removed, mixed with 40 µL 2× SDS-PAGE sample buffer, boiled at 95 °C for 10 min, and stored at −20 °C for western blot analysis. The remaining sample extract was incubated with 20 µL GFP-Trap-M magnetic beads (Chromotek; beads were prewashed three times with 500 µL ice cold wash buffer [GTEN with 1 mM PMSF]) on a rotary mixer for 1 h at 4 °C. Beads were magnetically separated from the sample supernatant and washed three times with 500 µL ice cold wash buffer; then, they were resuspended in 50 µL 2× SDS-PAGE sample buffer and boiled at 95 °C for 10 min. The resulting samples were separated by SDS-PAGE and analyzed by immunoblotting as above.

### Confocal Imaging.

Leaf cells from *N. benthamiana* were imaged as described (72) at 2 dpi using a Zeiss 710 microscope with Zeiss PL APO 403/1.0 water dipping objectives. GFP was excited at 488 nm with an argon laser, and emissions were collected at 500 to 530 nm. Monomeric red fluorescent protein (mRFP) was excited using a 561-nm line diode laser with emissions collected at 600 to 630 nm. The pinhole was set at 1 airy unit. Single optical slices and *z* stacks were collected from cells expressing low levels of protein fusions to minimize potential artifacts. Images were processed using the Zen 2010 software.

### Supplemental Information.

*SI Appendix* includes *SI Appendix*, Figs. S1–S14 and Dataset S1–S6.

## Supplementary Material

Supplementary File

Supplementary File

Supplementary File

Supplementary File

Supplementary File

Supplementary File

Supplementary File

## Data Availability

All study data are included in the article and/or supporting information.
