# Supplementary material for: Exploiting breakdown in nonhost effector–target interactions to boost host disease resistance
Source: Proc Natl Acad Sci U S A. 2022 Aug 22;119(35):e2114064119. doi: 10.1073/pnas.2114064119 (PMC9436328; doi:10.1073/pnas.2114064119)
Supplement: Supplementary File [file pnas.2114064119.sapp.pdf]

Supplemental Information

Exploiting breakdown in nonhost effector-target interactions to boost host disease resistance

Hazel McLellan[1§], Sarah E Harvey[2,3§], Jens Steinbrenner[3,4§], Miles R Armstrong[1], Qin He[1,5,6], Rachel Clewes[3], Leighton Pritchard[7,8], Wei Wang[1], Shumei Wang[1,9], Thomas Nussbaumer[10], Bushra Dohai[10], Qingquan Luo[4,11], Priyanka Kumari[4], Hui Duan[12], Ana Roberts[3], Petra C Boevink[5], Christina Neumann[4], Nicolas Champouret[12], Ingo Hein[1,5], Pascal Falter-Braun[10], Jim Beynon[3], Katherine Denby[2,3*] & Paul RJ Birch[1,5*]

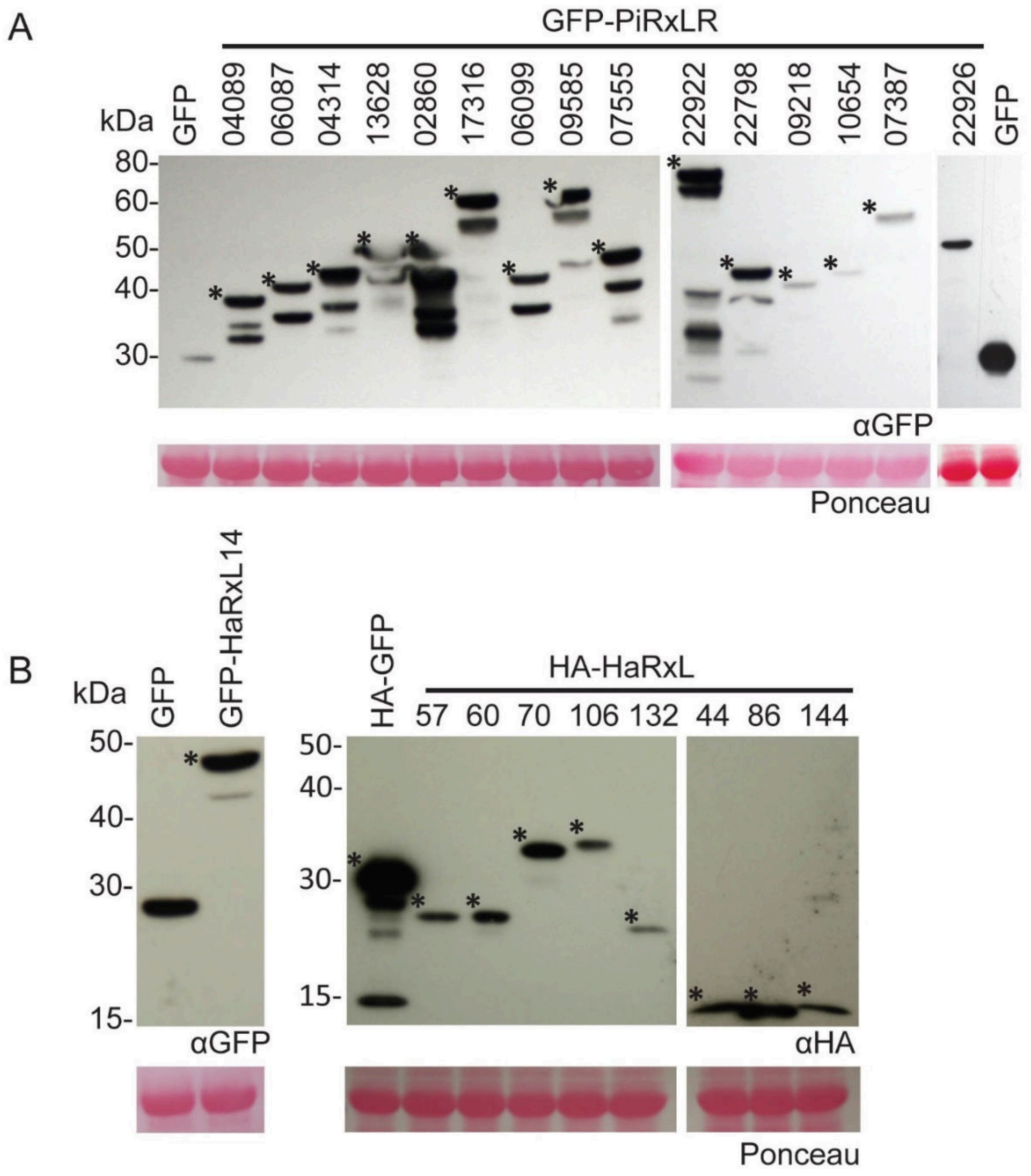

Figure S1: Stability of effector fusions in *N. benthamiana*.

Immunoblots show protein stability of (A) GFP-PiRxLR effectors and (B) GFP- or HA-HaRxLR effectors when expressed transiently in *Nicotiana benthamiana* using *Agrobacterium*-mediated expression. Protein loading is shown using Ponceau staining and the expected sized bands in Kilodaltons are indicated with asterisks.

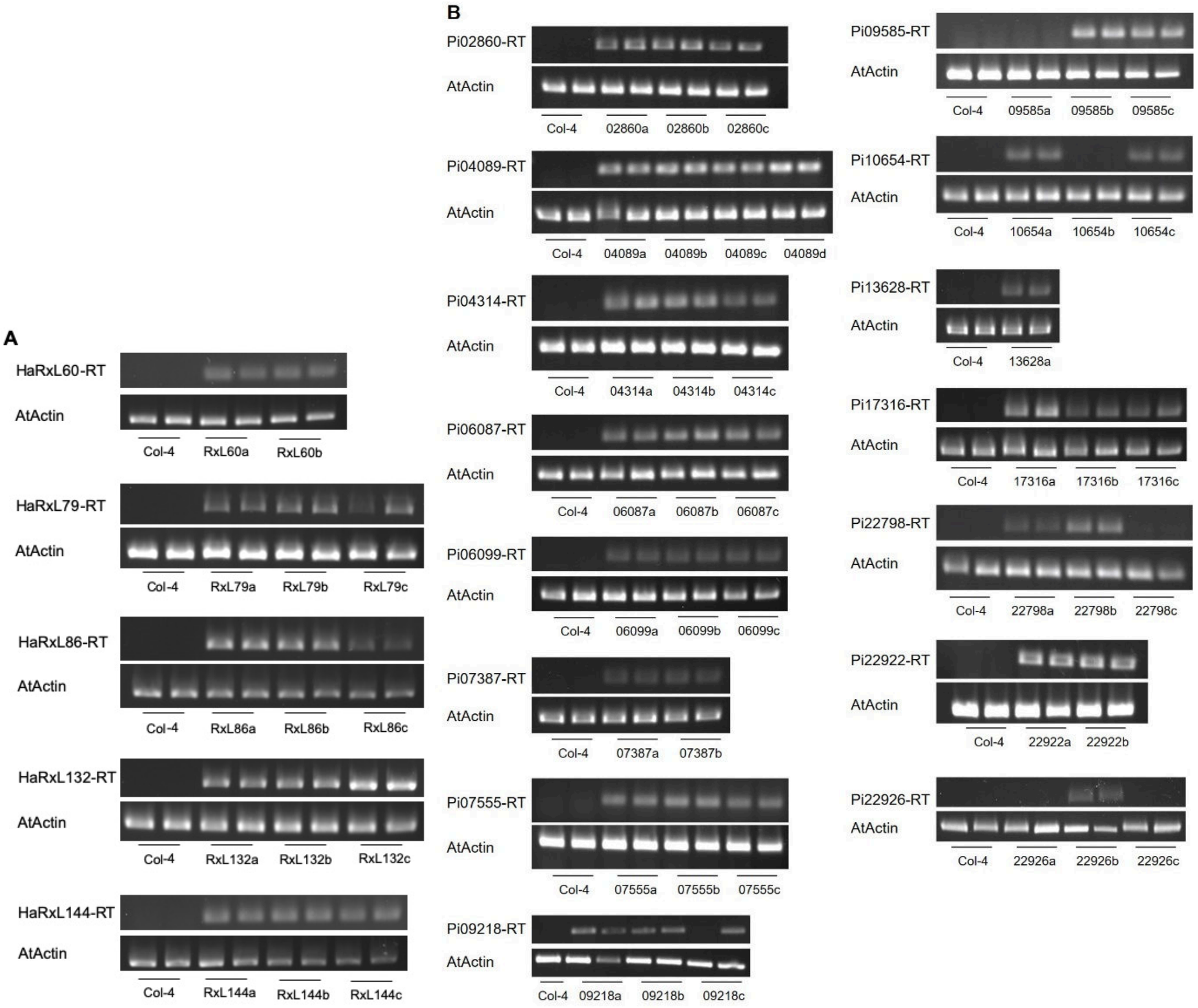

Figure S2: Expression of effectors in Arabidopsis transgenic lines.

Semi-quantitative RT-PCR showing the detection of expression of (A) HaRxLR effectors and (B) PiRxLR effectors in transgenic Arabidopsis plant lines. Col-4 is used as a negative control and *AtActin* is used as a housekeeping gene to show cDNA presence and quality. The remaining HaRxL lines are those used in Fabro et al., (2011).

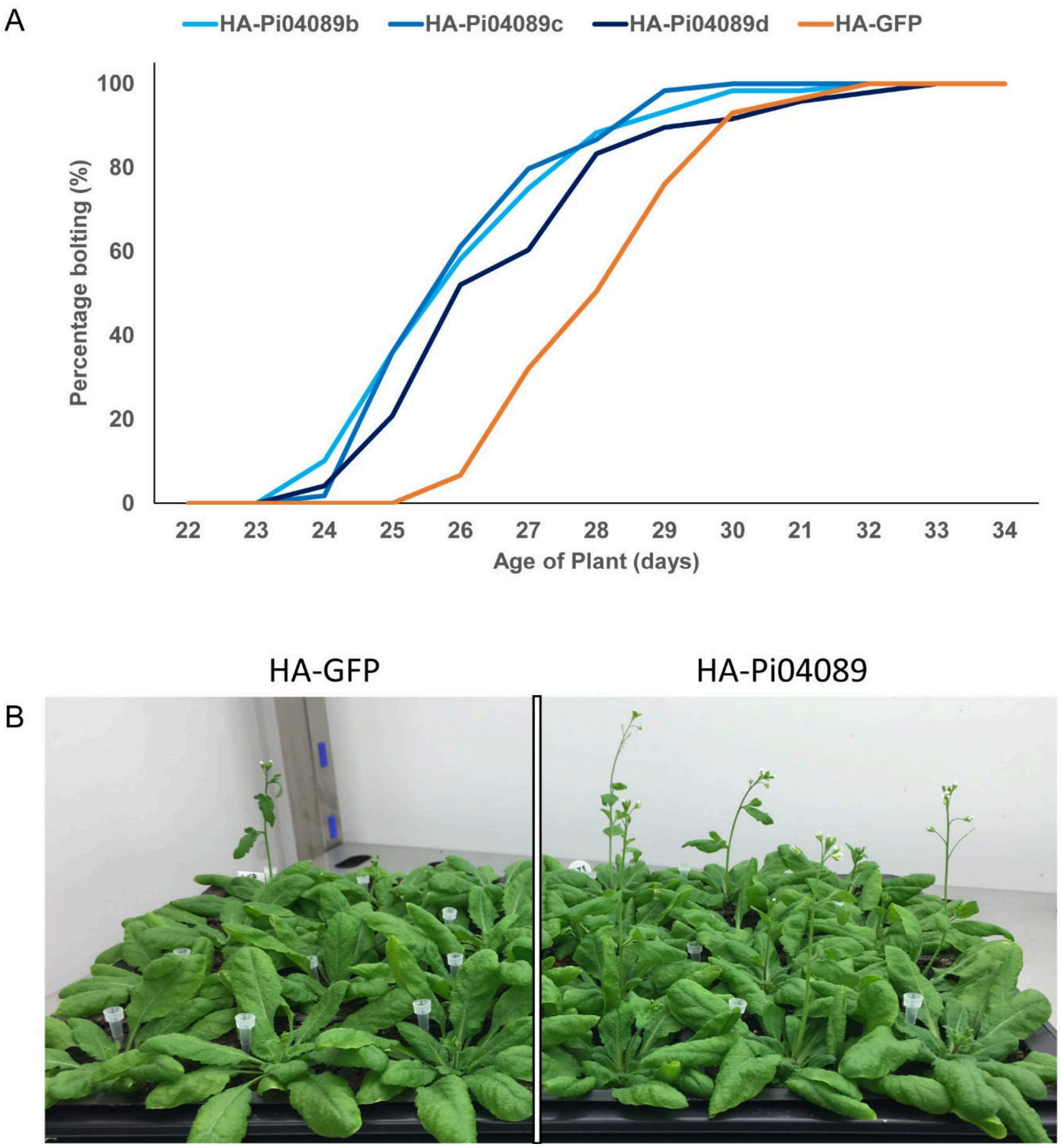

Figure S3: Arabidopsis transgenic lines expressing HA-Pi04089 have an early flowering phenotype.

(A) Graph shows the changes in the percentage of plants bolting between 21 to 34 days old. Transgenic lines expressing HA-GFP are used as controls. (B) Images showing early bolting phenotype of HA-Pi04089 transgenic Arabidopsis compared to the HA-GFP control.

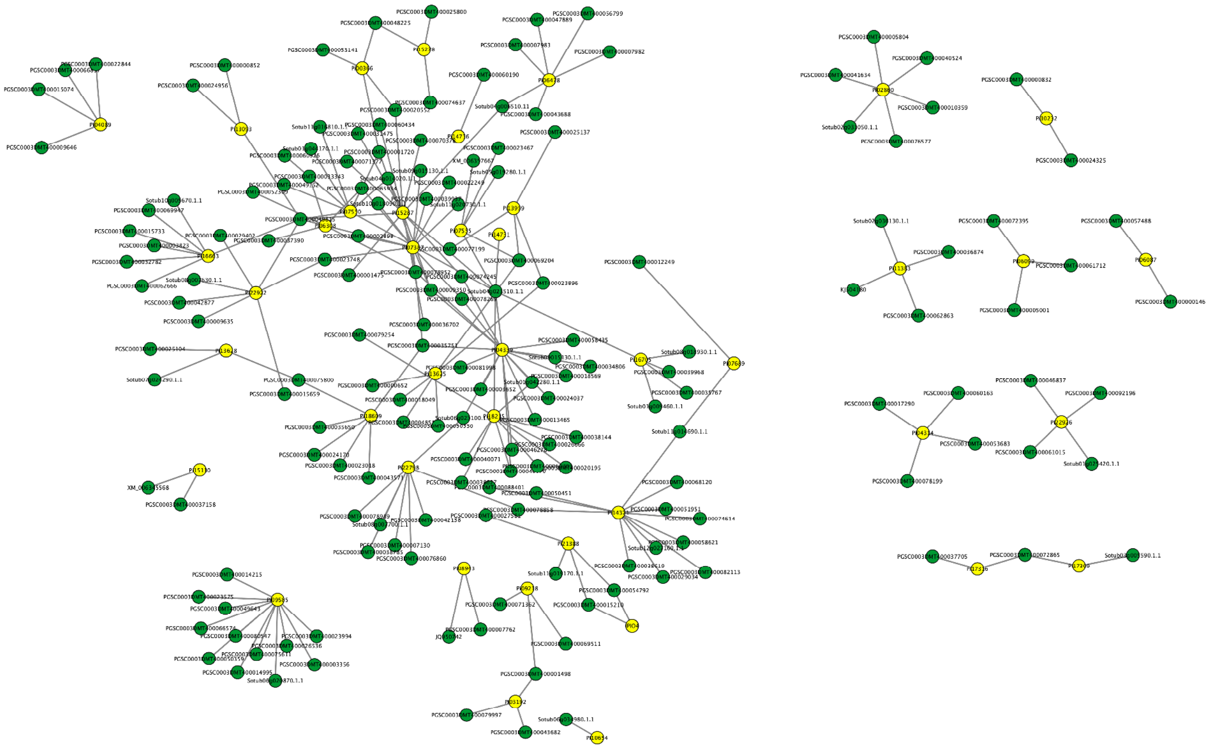

Figure S4: PiRxLR cY2H interaction network in Potato

High resolution network diagram representation of the cY2H interaction network of 64 PiRxLR effectors (yellow circles) with 169 potato candidate target proteins (green circles). Straight edges indicate 215 protein-protein interactions (PPIs). Each effector and target is annotated with gene accession numbers and identifiers as indicated in Dataset S2.

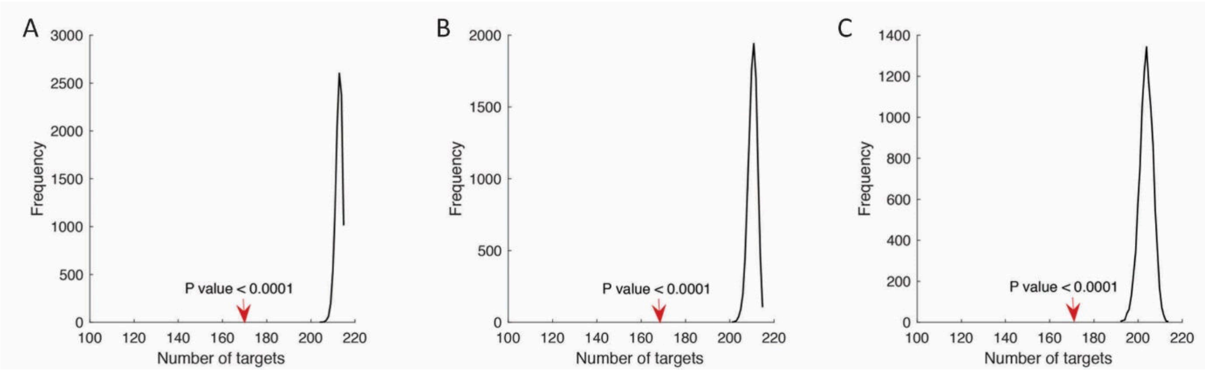

Figure S5. Convergence analysis.

Graphs plot the frequency versus the number of unique targets. The y-axis represents the frequency of obtaining, randomly, a unique number of targets (x-axis) within 10,000 iterations. Three search spaces were used: (A)10,000; (B) 5,000; and (C) 2,000 proteins. The actual observed value (n=169) is shown with a red arrow.

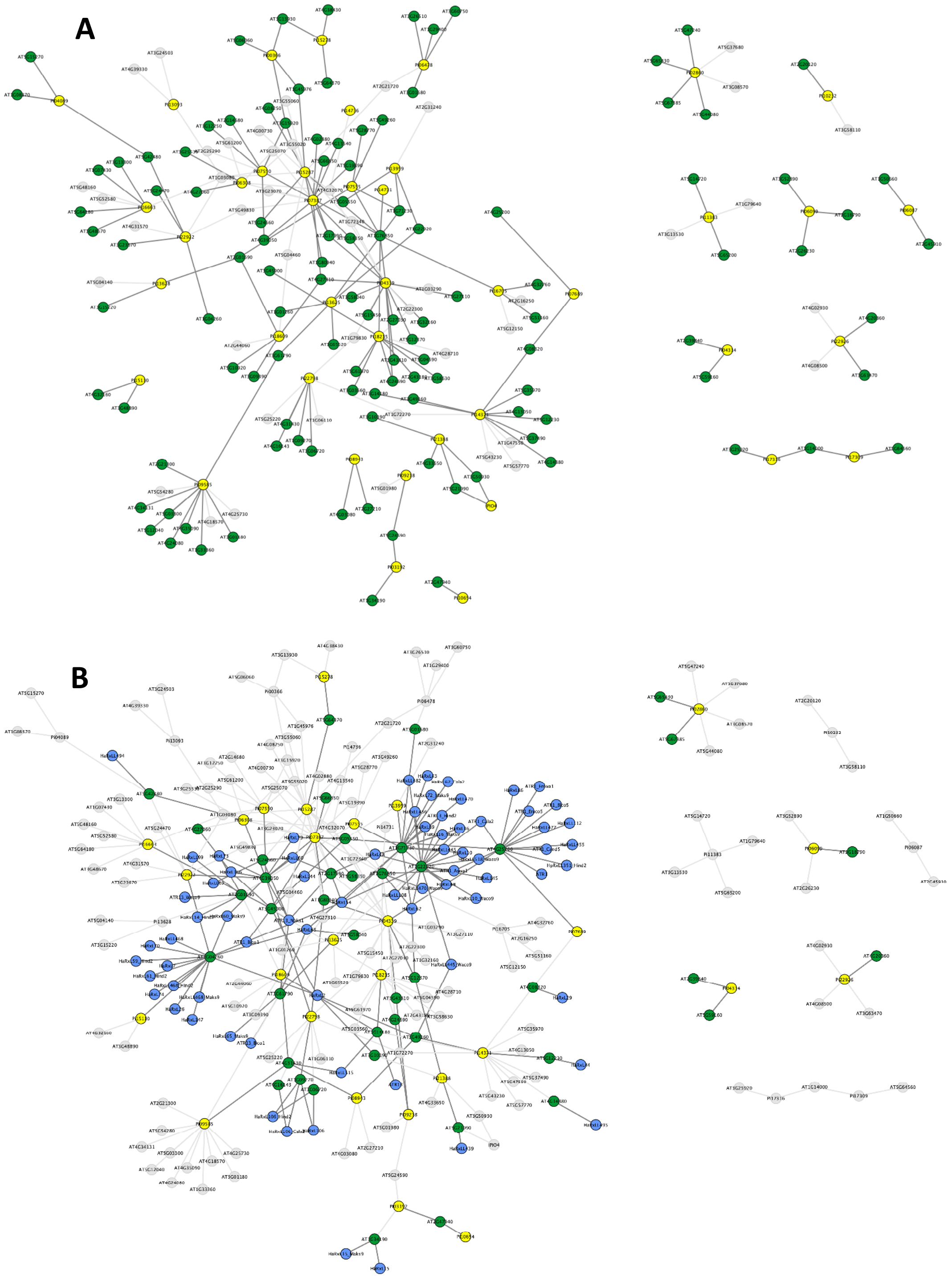

Figure S6: High resolution network diagrams of the candidate cAtOrth MoY2H screen.

(A) Network diagram representation of the cAtOrths (green circles) of potato cY2H targets which were identified and cloned.

(B) Network diagram representing the newly identified PPIs detected between HaRxLs and cAtOrths.

PiRxLRs (Yellow circles), cAtOrths (green circles) HaRxLs (Blue circles). Straight edges indicate (PPIs) and greyed out circles and edges indicate candidate orthologues which were not cloned and untestable interactions respectively. Each effector and target are annotated with gene accession numbers and identifiers as indicated in Dataset S3.

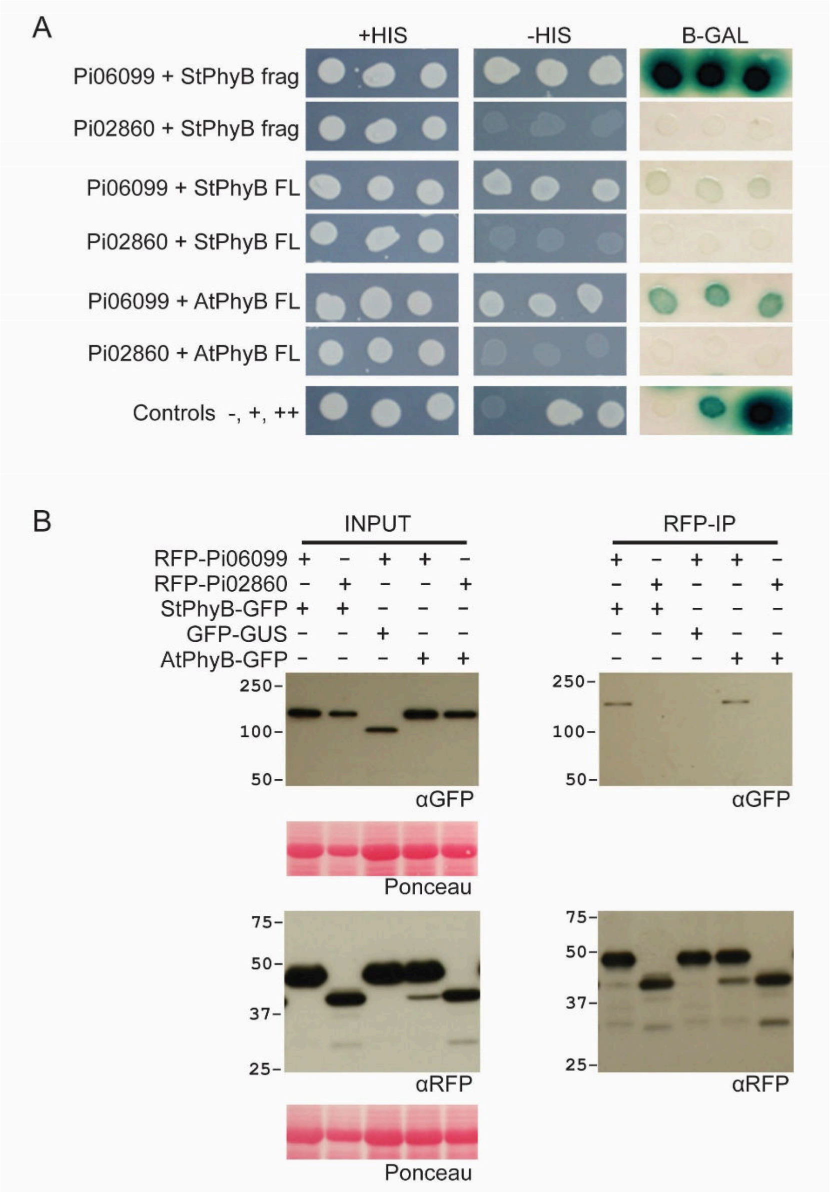

Figure S7: Pi06099 interacts with full length Phytochrome B from Potato and Arabidopsis.

(A) Yeast containing Pi06099 and StPhyB fragment (frag; Dataset S3) and full length (FL) and AtPhyB FL grew on medium lacking histidine (-HIS) and showed β-galactosidase (BGAL) activity indicating protein-protein interaction. Yeast co-expressing control effector Pi02860 do not activate any reporter genes with any Phytochrome B construct. All yeast grew on medium containing histidine (+HIS). The yeast controls are as follows: - = no interaction, + = weak interaction, ++= strong interaction.

(B) Co-immunoprecipitation assays confirmed the interaction *in planta*. Following pull downs with RFP-trap beads; StPhyB-GFP and AtPhyB-GFP associated with RFP-Pi06099 but not RFP-Pi02860. Control GFP-GUS did not associate with RFP-Pi06099. Expression of constructs in *N. benthamiana* leaves is indicated by a "+." Protein size markers are indicated in kilodaltons, and protein loading is indicated by Ponceau stain.

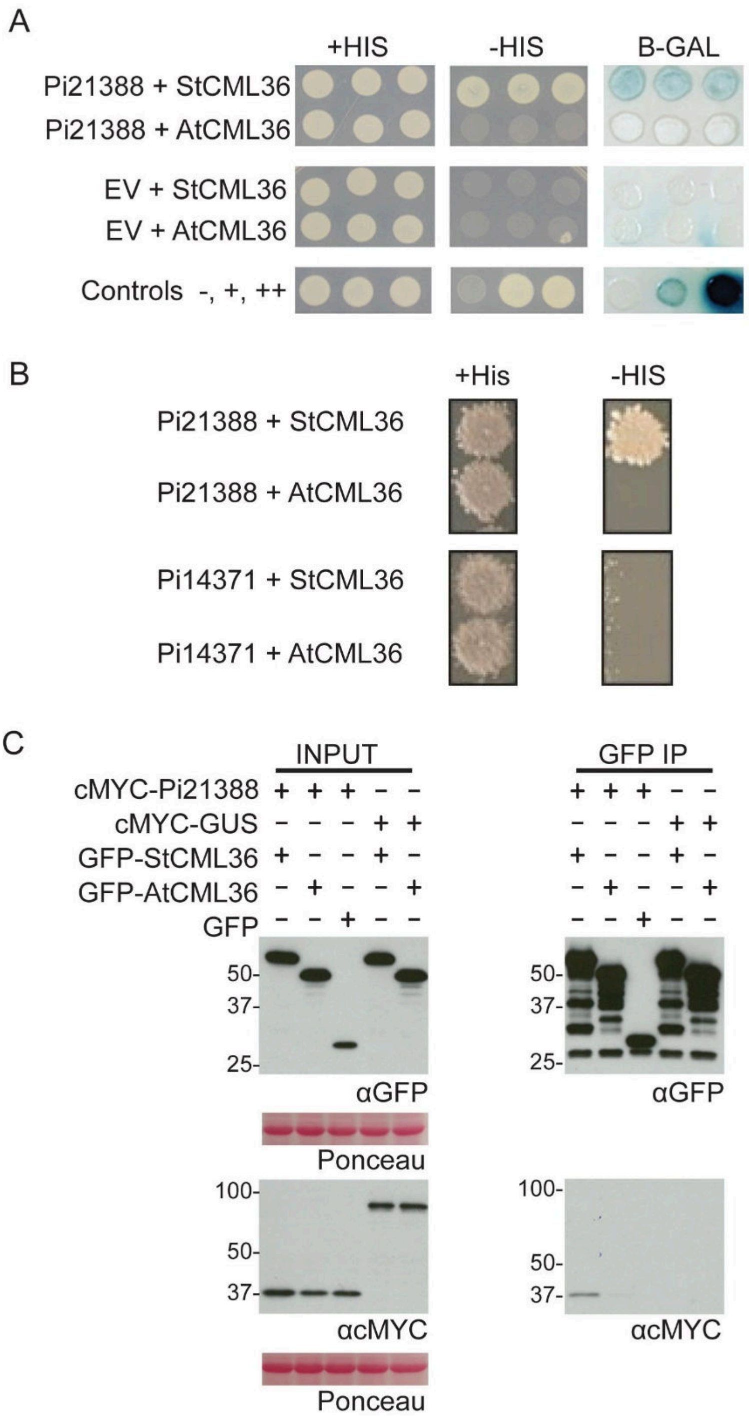

Figure S8: Pi21388 interacts in yeast with potato but not Arabidopsis CML36.

(A) Yeast containing Pi21388 (ipiO1/AvrBlb1) and StCML36 grew on medium lacking histidine (-HIS) and showed β-galactosidase (BGAL) activity indicating protein-protein interaction whereas, yeast containing Pi21388 and AtCML36 did not. Yeast co-expressing the empty vector (EV) control and either of the CML36 constructs do not activate reporter genes. All yeast grew on medium containing histidine (+HIS). The yeast controls are as follows: - = no interaction, + = weak interaction, ++= strong interaction.

(B) In the mating-based Y2H system used for MoY2H screens, Yeast containing Pi21388 and StCML36 but not Pi21388 and AtCML36 grew on medium lacking histidine (-HIS). Yeast containing effector Pi14371 and either StCML36 or AtCML36 did not grow on medium lacking histidine. All yeast grew on medium containing histidine (+HIS).

(C) Co-immunoprecipitation assays confirmed the interaction *in planta*. Following pull downs with GFP-trap beads; GFP-StCML36 associated with cMYC-Pi21388 but free GFP did not. However, GFP-AtCML36 only very weakly associated with cMYC-Pi21388. Control cMYC-GUS did not associate with GFP-CML36 from potato or Arabidopsis. Expression of constructs in *N. benthamiana* leaves is indicated by a "+." Protein size markers are indicated in kilodaltons, and protein loading is indicated by Ponceau stain, all clones of StCML36 and AtCML36 used are full length in both Y2H and CoIP.

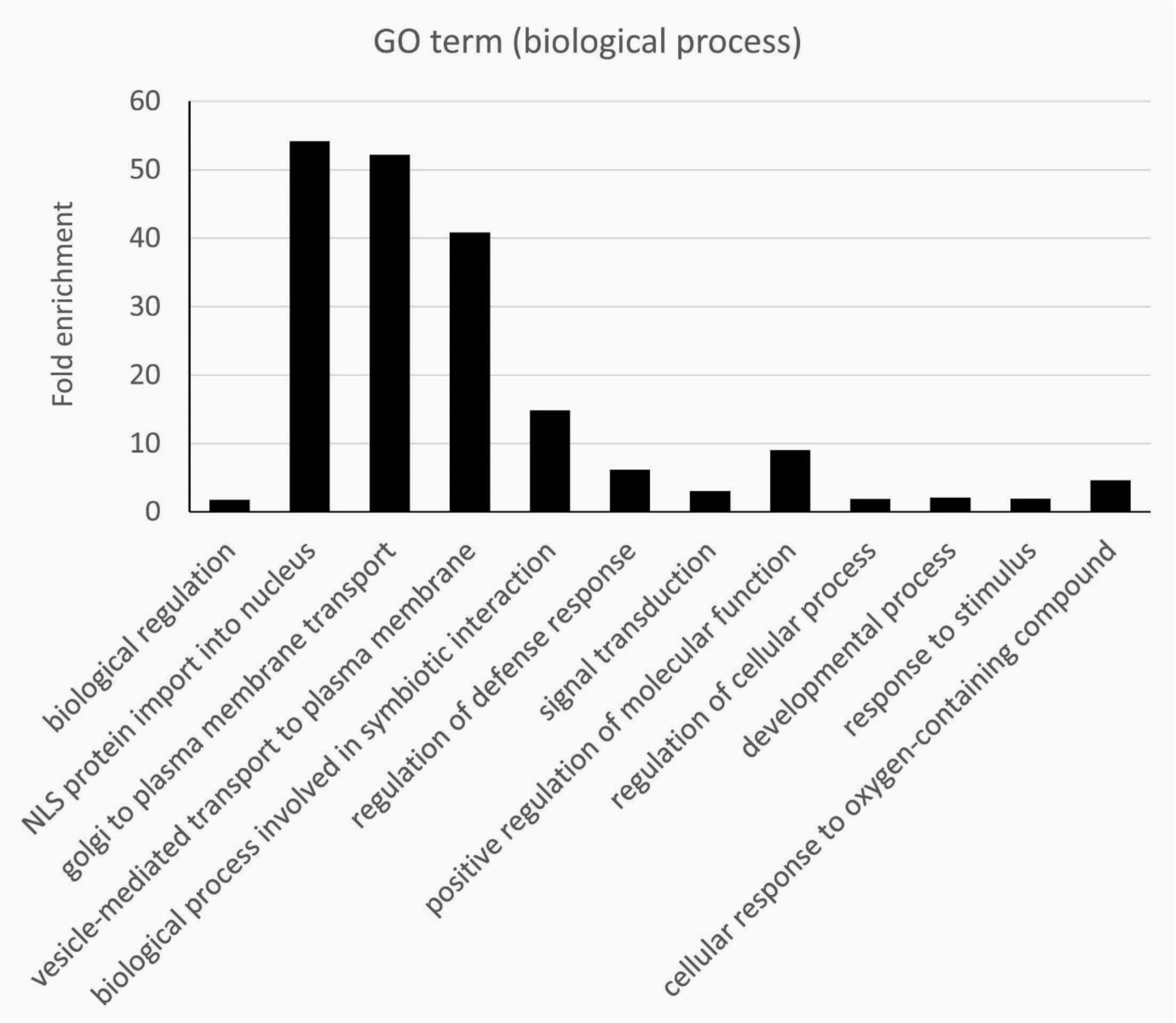

Figure S9: GO term analysis of biological processes for the cAtOrths.

Graph shows the fold enrichment GO terms for the top 12 biological processes for the cAtOrths.

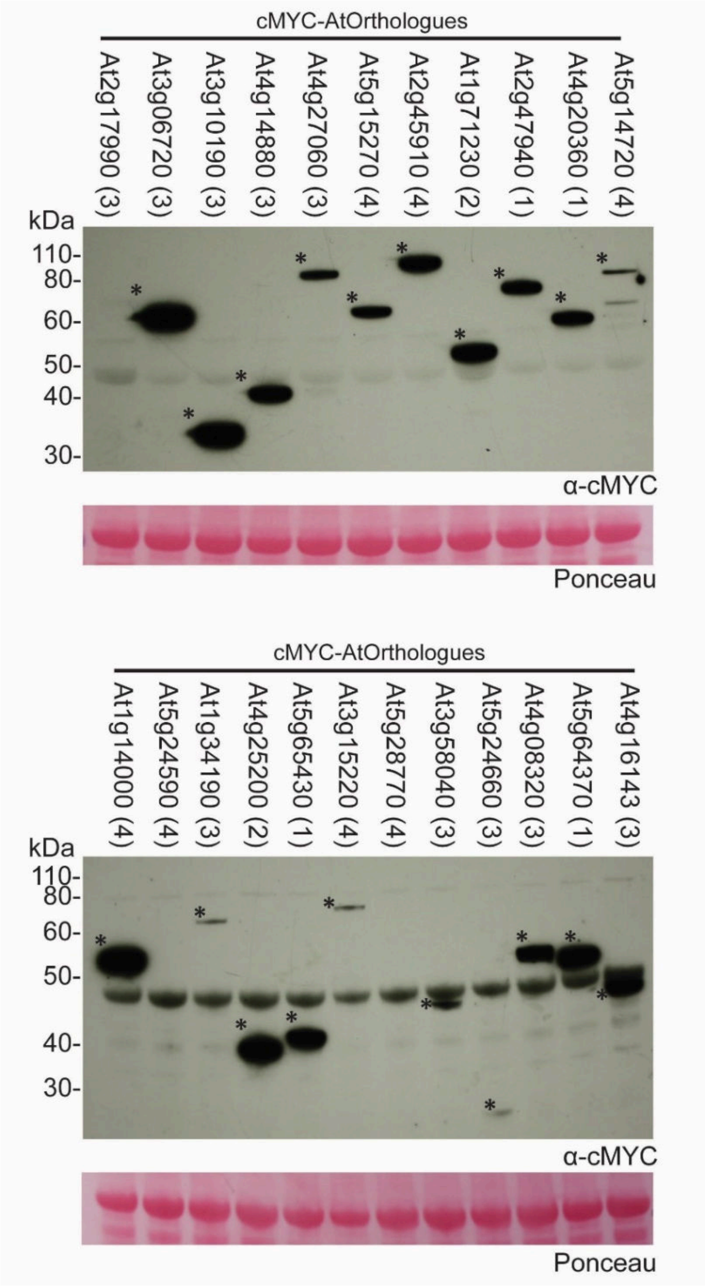

Figure S10: Stability of cAtOrth protein fusions in *N. benthamiana*.

Immunoblots show protein stability of cMyc-tagged cAtOrths when expressed transiently in *N. benthamiana* using *Agrobacterium* mediated expression. Protein loading is shown using Ponceau staining and the expected sized bands in kilodaltons are indicated with asterisks. The category of interaction for each cAtOrth is shown in brackets alongside the gene name. Protein expression was not detectable for At2g17990, At5g24590 or At5g28770.

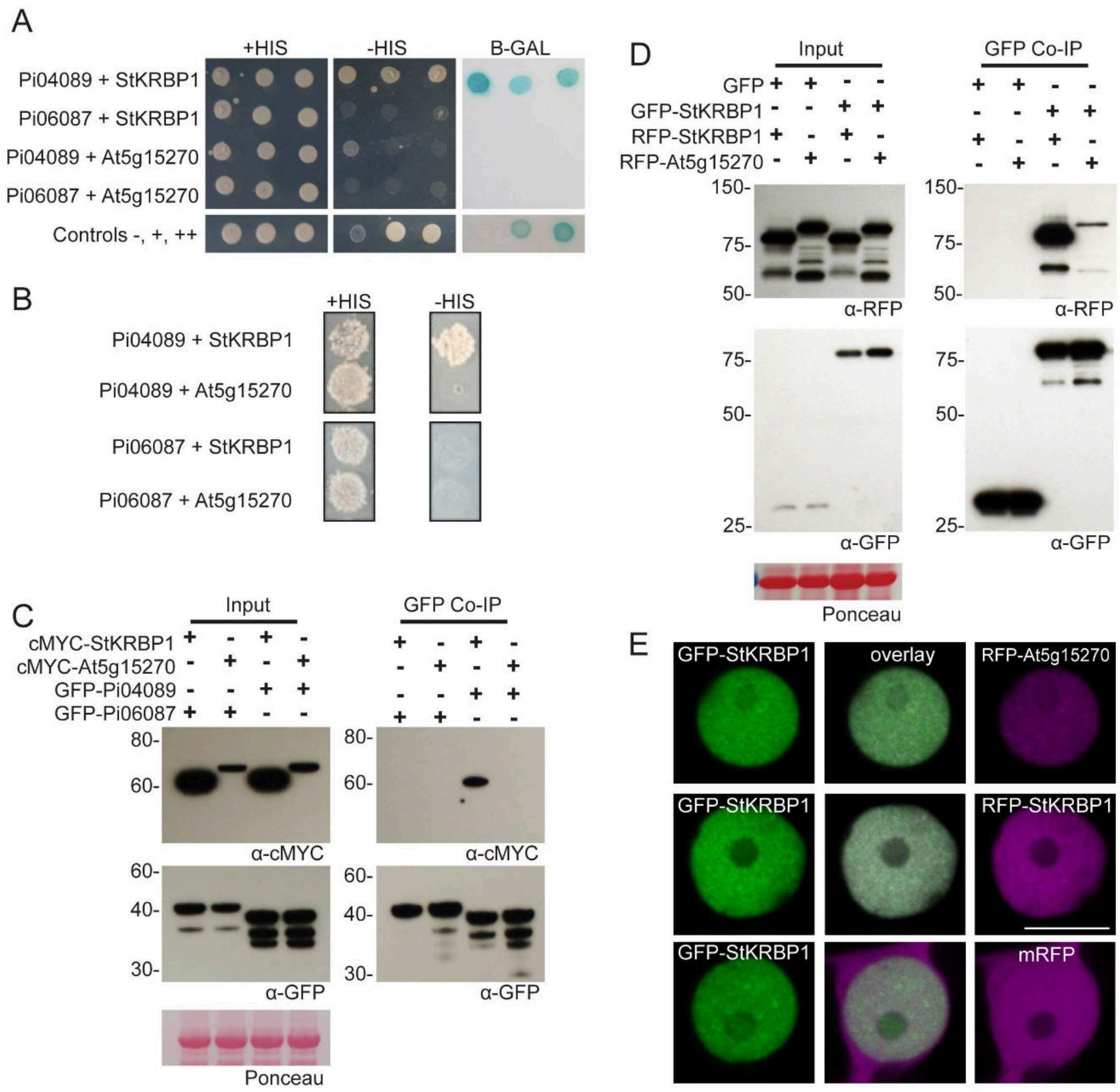

Figure S11: Pi04089 interacts with the potato protein StKRBP1 but not its candidate cAtOrth (At5g15270).

(A) Yeast containing Pi04089 and StKRBP1 grew on medium lacking histidine (-HIS) and showed β-galactosidase (BGAL) activity indicating protein-protein interaction. Yeast co-expressing Pi04089 and At5g15270 do not activate any reporter genes, nor does the effector control Pi06089 when expressed with either StKRBP1 or At5g15270. All yeast grew on medium containing histidine (+HIS). The yeast controls are as follows: - = no interaction, + = weak interaction, ++= strong interaction.

(B) In the mating-based Y2H system used for MoY2H screens, yeast containing Pi04089 and StKRBP1 but not Pi04089 and At5g15270 grew on medium lacking histidine (-HIS). Yeast

containing Pi06087 and either StKRBP1 or At5g15270 did not grow on medium lacking histidine. All yeast grew on medium containing histidine (+HIS).

(C) Co-immunoprecipitation assays confirmed the interaction *in planta*. Following pull downs with GFP-trap beads; GFP-Pi04089 associated with cMYC-StKRBP1 but not cMYC-At5g15270, whereas control GFP-Pi06089 interacted with neither. Expression of constructs in *N. benthamiana* leaves is indicated by a "+." Protein size markers are indicated in kilodaltons, and protein loading is indicated by Ponceau stain.

(D) Co-immunoprecipitation assays confirmed the interaction *in planta*. Following pull downs with GFP-trap beads; GFP-StKRBP1 associated with RFP-StKRBP1 and also weakly with RFP-At5g15270. whereas, free GFP control immunoprecipitated neither. Expression of constructs in *N. benthamiana* leaves is indicated by a "+." Protein size markers are indicated in kilodaltons, and protein loading is indicated by Ponceau stain.

(E) Confocal images show that GFP-StKRBP1 colocalises to nuclear speckles with RFP-StKRBP1 and RFP-At5g15270 but not with free mRFP. Scale bar is 10µm.

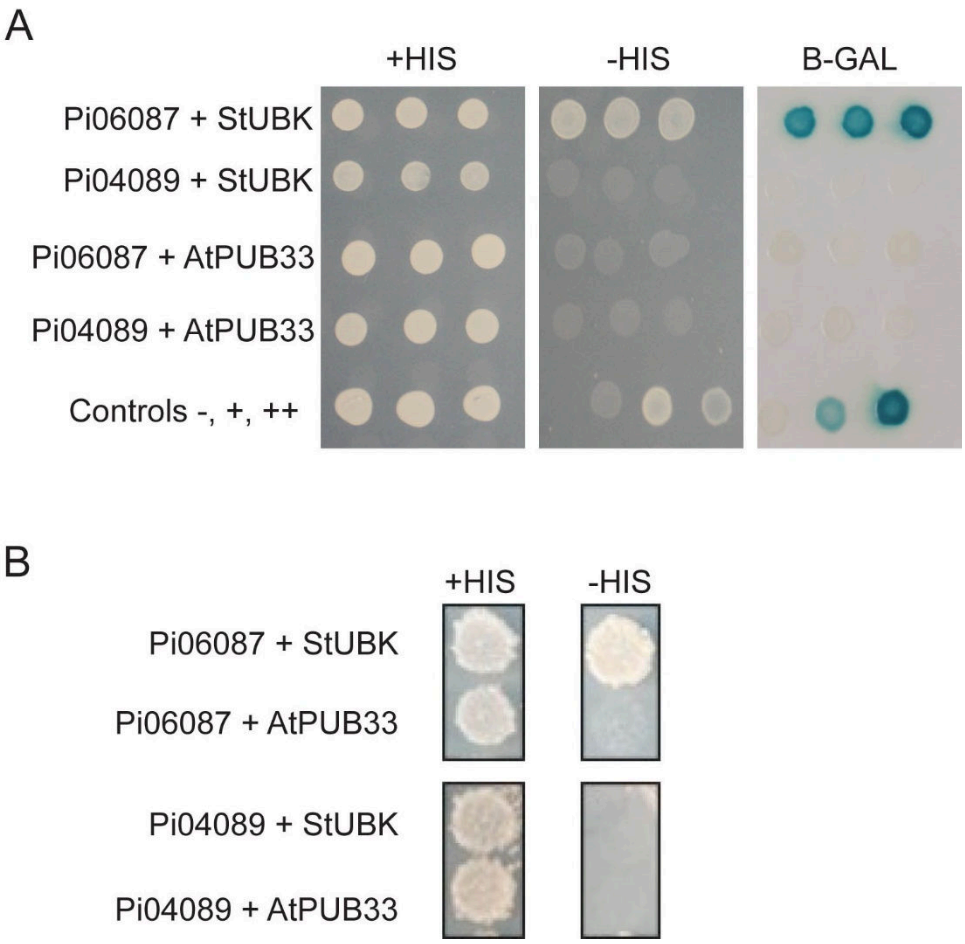

Figure S12: Pi06087 interacts with StUBK but not its cAtOrth AtPUB33.

(A) Yeast containing Pi06087 and StUBK grew on medium lacking histidine (-HIS) and showed β-galactosidase (BGAL) activity indicating protein-protein interaction. Yeast co-expressing Pi06087 and AtPUB33 do not activate any reporter genes, nor does the effector control Pi04089 when expressed with either StUBK or AtPUB33. All yeast grew on medium containing histidine (+HIS). The yeast controls are as follows: - = no interaction, + = weak interaction, ++= strong interaction.

(B) In the mating-based matrix Y2H system used for MoY2H screens, yeast containing Pi06087 and StUBK but not Pi06087 and AtPUB33 grew on medium lacking histidine (-HIS). Yeast containing Pi04089 and either StUBK or AtPUB33 did not grow on medium lacking histidine. All yeast grew on medium containing histidine (+HIS).

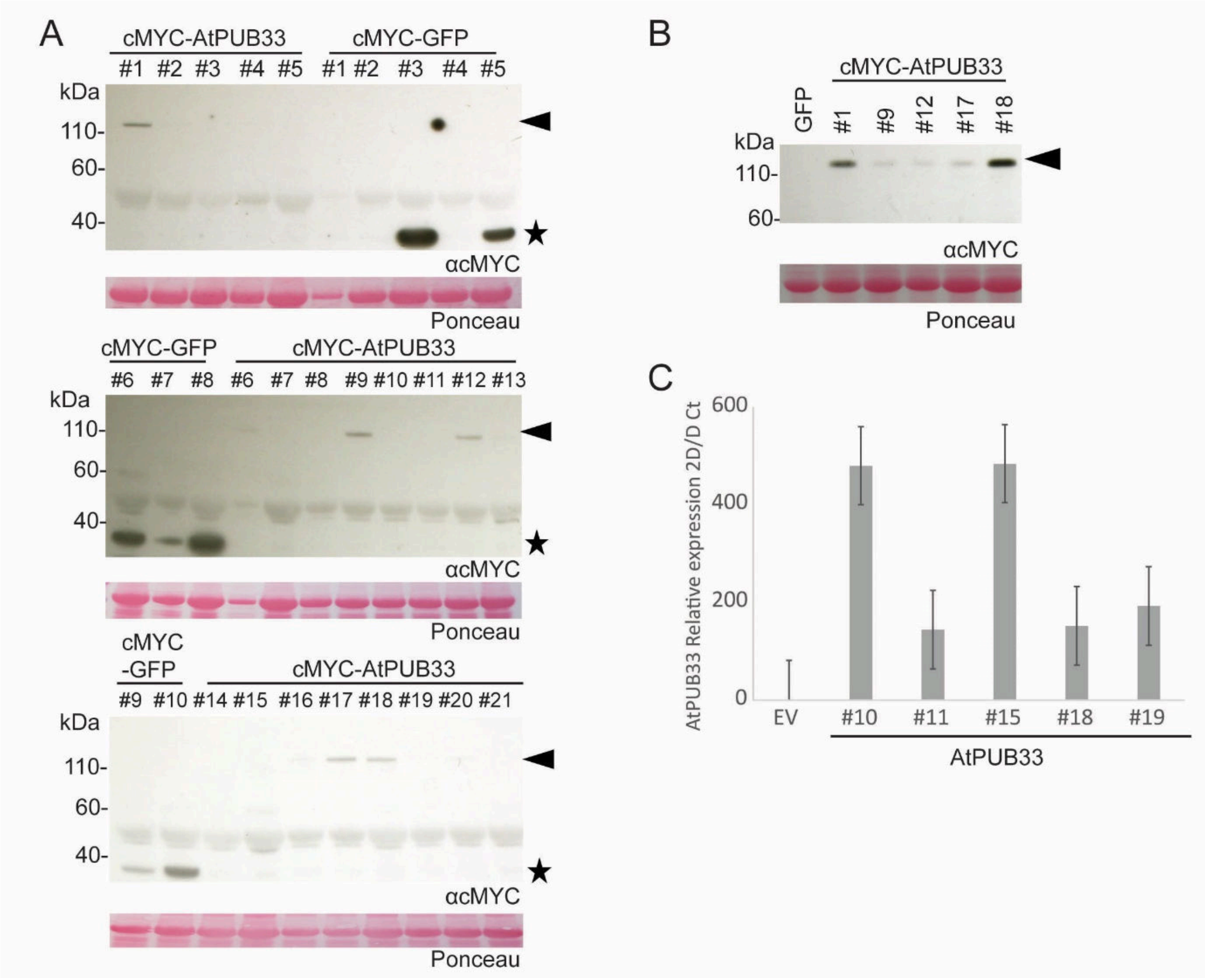

Figure S13: Transgenic line confirmation and selection.

(A) T_0 *N. benthamiana* transformants which were selected on media containing kanamycin were tested for the presence of cMYC-AtPUB33 (arrow head) or cMYC-GFP (star) by immunoblotting. For cMYC-AtPUB33 lines #1,9,12,17 and 18 showed detectable full length fusion protein expression. For cMYC-GFP control lines #3,5,6,7,8,9 and 10 had protein expression.

(B) Immunoblot shows T_2 *N. benthamiana* cMYC-AtPUB33 transgenic lines with detectable full length fusion protein indicated with an arrow head. Ponceau stain shows protein loading. Size ladder is in kilodaltons.

(C) Graph of qRT-PCR data showing detectable *AtPUB33* expression in five individual *AtPUB33* potato overexpression transgenic lines compared to plants transformed with the empty transformation vector as a control. Error bars are standard error.

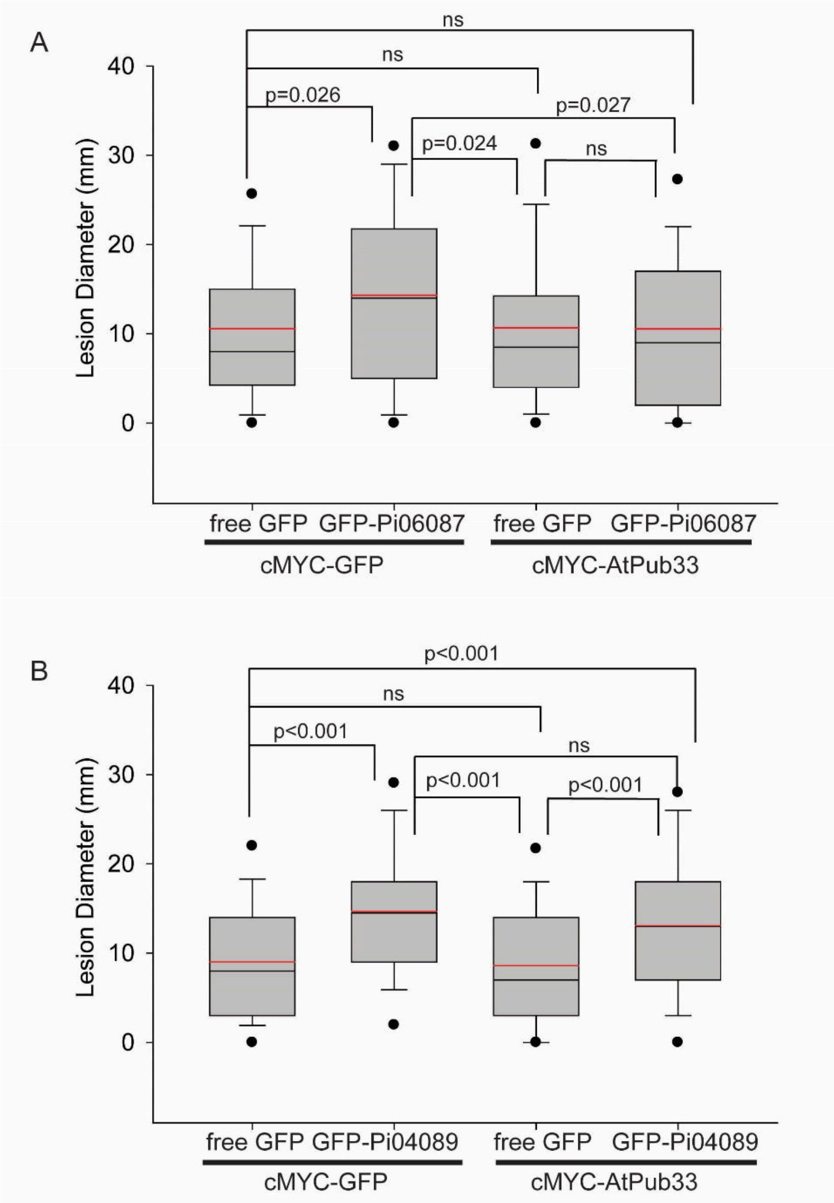

Figure S14: Pi06087 is unable to enhance *P. infestans* colonisation on AtPUB33 transgenic *N. benthamiana*.

Boxplot shows *P. infestans* lesion diameter in stable *N. benthamiana* transgenic lines overexpressing (A) cMYC-GFP or cMYC-AtPUB33 which are then overexpressing either free GFP or GFP-Pi06087 by transient agro-expression. GFP-Pi06087 can significantly enhance *P. infestans* colonisation on cMYC-GFP plants but not on cMYC-AtPUB33 plants.

Boxplot shows *P. infestans* lesion diameter in stable *N. benthamiana* transgenic lines overexpressing (B) cMYC-GFP or cMYC-AtPUB33 which are then overexpressing either free GFP or GFP-Pi04089 by transient agro-expression. GFP-Pi04089 can significantly enhance *P. infestans* colonisation on both cMYC-GFP and cMYC-AtPUB33 plants.

Boxplots show combined data from 5 independent replications of the experiments (n=88). The median is shown with a black line, the mean is indicated with a red line and boxes show the interquartile ranges (IQR). Filled circles indicate the 5th and 95th percentile outliers. Whiskers indicate 1.5 x IQR. P values indicate if there are significant differences as tested by one way ANOVA with pairwise comparisons performed using the Holm-Sidak method, ns= non-significant.

Dataset S1: RXLR effector sequence information.

(S1A) PiRXLR nucleotide and protein sequences and primer information.

(S1B) HaRXLR nucleotide and protein sequence information.

Dataset S2: PiRXLR-potato cY2H screens.

(S2A) Summary of the interactors from 64 PiRXLR Y2H screens.

(S2B) Effectors with shared candidate targets.

(S2C) PiRXLR family percentage shared target breakdown.

Dataset S3: Summary of the cAtOrth MoY2H screen.

Dataset S4: Comparisons with previous matrix Y2H assays by Mukhtar *et al* (13) and Weβling *et al* (14).

Dataset S5: cAtOrth sequence information.

Dataset S6: Additional primer sequences.
